# Influence of muscle groups’ activation on proximal femoral growth tendency

**DOI:** 10.1007/s10237-017-0925-3

**Published:** 2017-06-22

**Authors:** Priti Yadav, Sandra J. Shefelbine, Eva Pontén, Elena M. Gutierrez-Farewik

**Affiliations:** 1 KTH Engineering Sciences, Mechanics, Royal Institute of Technology, Stockholm, Sweden; 2 KTH BioMEx Center, Royal Institute of Technology, Stockholm, Sweden; 3 Department of Women’s and Children’s Health, Pediatric Orthopaedic Surgery, Karolinska Institutet, Stockholm, Sweden; 4 Department of Mechanical and Industrial Engineering, Northeastern University, Boston, USA

**Keywords:** Bone tissue modeling, Deformity development, Biomechanics, Individualized, Finite element analysis, Osteogenic index

## Abstract

Muscle and joint contact force influence stresses at the proximal growth plate of the femur and thus bone growth, affecting the neck shaft angle (NSA) and femoral anteversion (FA). This study aims to illustrate how different muscle groups’ activation during gait affects NSA and FA development in able-bodied children. Subject-specific femur models were developed for three able-bodied children (ages 6, 7, and 11 years) using magnetic resonance images. Contributions of different muscle groups—hip flexors, hip extensors, hip adductors, hip abductors, and knee extensors—to overall hip contact force were computed. Specific growth rate for the growth plate was computed, and the growth was simulated in the principal stress direction at each element in the growth front. The predicted growth indicated decreased NSA and FA (of about $0.1 {^{\circ }}$ over a four-month period) for able-bodied children. Hip abductors contributed the most, and hip adductors, the least, to growth rate. All muscles groups contributed to a decrease in predicted NSA ($\sim$0.01${^{\circ }}$–0.04${^{\circ }})$ and FA ($\sim$0.004${^{\circ }}$–$0.2{^{\circ }}$), except hip extensors and hip adductors, which showed a tendency to increase the FA ($\sim$0.004${^{\circ }}$–$0.02{^{\circ }}$). Understanding influences of different muscle groups on long bone growth tendency can help in treatment planning for growing children with affected gait.

**Electronic supplementary material:**

The online version of this article (doi:10.1007/s10237-017-0925-3) contains supplementary material, which is available to authorized users.

## Introduction

The longitudinal growth of long bones is due to the endochondral ossification process that takes place at growth plates, located at the ends of the long bones. There are multiple factors, such as hormones, nutrients, genetics and mechanical loading that regulate the endochondral ossification process. Hormones, nutrients and genetics account for most of the overall bone growth, but mechanical loading regulates growth at the local level, influencing bone morphology (Narváez-Tovar and Garzón-Alvarado 2012).

The prevailing theory for mechanically modulated bone growth modeling states that endochondral growth and ossification are accelerated by cyclic octahedral shear stress and retarded by cyclic hydrostatic compressive stress (Carter et al. 1987). The mechanobiological contribution to the growth rate has been defined as the osteogenic index, which is the linear sum of maximum octahedral shear stress and minimum hydrostatic stress within the growth plate cartilage during a load cycle (Stevens et al. 1999).

The osteogenic index has been used to investigate the involvement of muscle and joint contact forces in the formation of increased neck shaft angle (NSA) in developmental hip dysplasia (Shefelbine and Carter 2004a) and to predict femoral anteversion (FA) in cerebral palsy (Shefelbine and Carter 2004b). It has been reported that abnormal muscle and joint contact forces in cerebral palsy may lead to the development of bone deformities such as increased NSA and FA (Carriero et al. 2011).

The osteogenic index estimates the mechanical growth *rate* but does not describes the resulting growth *direction* (Lin et al. 2009). Previous work has assumed growth in the deformation direction (Shefelbine and Carter 2004b; Carriero et al. 2011) based on the observational evidence *in vivo* for static loading (Arkin and Katz 1956). However, there is no literature found that supports this finding for other physiological load conditions. In the literature, it has also been reported that in order to avoid shear stress between the metaphysis and epiphysis, the growth plate topology tends to lie parallel to maximum principal stress in the region of minimum principal stresses and vice versa (Smith 1962; Ogden 2000). A numerical simulation of bone growth for an able-bodied child in the principal stress direction predicted the expected neck shaft and anteversion angle changes better than the growth in the deformation direction (Yadav et al. 2016).

Both osteogenic index and growth direction are dependent on the mechanical forces acting on the bone. The maximum mechanical forces acting directly on the bones are due to muscle contraction (i.e., muscle forces) and bone-to-bone contact forces (i.e., joint contact forces). Further, the joint contact forces in turn depend on both muscle forces and ground reaction force (GRF). Muscle forces contribute directly to the joint contact force as well as indirectly by accelerating the center of mass (Pandy 2001; Anderson and Pandy 2003). It has been reported that during gait high joint contact forces acting at lower extremity joints are primarily due to muscle tension (Herzog et al. 2003). Muscle forces, therefore, are one of the strong determinants of joint contact force and hence of bone morphology in a growing child.

There are multiple reports with evidence that bone growth and morphology are influenced by muscle action (Sharir et al. 2011). For example, shortened bone was observed in chicken embryos when their muscles were paralyzed (Drachman and Sokoloff 1966). In children with idiopathic clubfoot, bone malformation has been attributed to imbalance in (Feldbrin et al. 1995) or reduction of muscle forces (Ippolito et al. 2009). The increased FA in children with cerebral palsy has been attributed to abnormal hip rotator muscle forces (Cibulka 2004). Spastic or contracted hamstrings are believed to be a cause of crouch gait in some cases (Baumann et al. 1980; Sutherland and Davids 1993) and may lead to bone deformity development through abnormal bone loading.

The muscles can individually or interactively influence bone growth and morphological changes. It is, however, a challenge to understand the role of different muscle groups to bone growth in growing children. The current study aims to analyze how different muscle groups’ activation during able-bodied children’s normal gait influences proximal femoral growth tendency in the femoral head region. This study will help to elucidate the dominance of different muscle groups on proximal femoral morphological characteristics.

## Methods

### Data collection

Three typically-developing children participated in this study. Three-dimensional gait analysis of the subjects was performed with an 8-camera motion analysis system (Vicon MX40, Oxford, UK) and two force platforms (Kistler, Winterthur, Switzerland). Thirty-five reflective 9-mm markers were attached to specific anatomical landmarks using a full-body model. The subjects were instructed to walk at a comfortable speed along a 10-m walkway.

For each subject, magnetic resonance images (MRIs) of the lower body in a neutral supine position were collected at T1 contrast, with frontal and transverse slices of 3 mm thickness (Ingenia 3.0T, Philips, Best, The Netherlands).

### Model construction

Subject-specific femur models for all three subjects were constructed using MRI data (Mimics, Materialise NV, Leuven, Belgium). A contour-based segmentation approach (3D LiveWire tool) in conjunction with a mask editing tool was used for MRI data segmentation. The segmented images were then used to generate 3D surface models of each femur. Regions of cortical bone and of proximal and distal trabecular bone were generated using transverse plane MRI data. The bone marrow was constructed as the volume constrained by the inner surface of the cortical bone and the inferior and superior surfaces (along the transverse plane) of the proximal and distal trabecular bone, respectively. To construct the 3D volume of the growth plate, the region of proximal trabecular bone viewed from the frontal plane MRIs was subtracted (Boolean operation) from the region of proximal trabecular bone viewed from the transverse plane MRI data. The smoothening techniques were used to smooth the rough edges or surfaces for better quality mesh generation (3-matic, Materialise NV, Leuven, Belgium).

The NSA and FA were measured from the 3D surface models (Yadav et al. 2016) and are described in Table 1. The growth plate thickness was determined by finding the minimum distance between points of the growth plate’s upper-most surface to the lower-most surface (MacDonald et al. 2000).

**Table 1:** Geometrical descriptions and subject measurements of proximal femoral morphology

	Age (year)	Weight (kg)	NSA (${^{\circ }})$	FA (${^{\circ }})$	Growth plate thickness (mm)	
					Min–max	Mean (SD)
Subject 1	6	20.7	140	34	2.7–4.6	4.1 (0.3)
Subject 2	7	23.8	136	33	4.5–7.4	5.9 (0.8)
Subject 3	11	49.5	138	18	2.4–4.8	3.6 (0.6)

*NSA* neck shaft angle, *FA* femoral anteversion

### Force computation

#### Musculoskeletal model

A generic musculoskeletal model (GaitModel, SIMM7.0, Musculographics Inc., Santa Rosa, CA, USA) was scaled based on collected marker positions during a static pose. The generic model consisted of 41 body segments, 41 joints, 88 lower limb musculotendon actuators (including 2 patellar ligaments) and 40 degrees of freedom The hip joint was modeled as a ball-and-socket joint. The knee and ankle joints were modeled as hinge joints. The scaled femur model was further modified to match each subject’s femur geometry (namely, NSA and FA) using a deform tool in SIMM7.0. To avoid any errors associated with the order of angular transformation, the modified-scaled femur model was verified against the subject’s MRI-based femur model by overlapping each other.

#### Muscles forces and hip contact force computation

Inverse dynamics analysis was performed for all three subjects using the modified-scaled musculoskeletal models to compute the joint moments. In this inverse dynamics analysis, the equations of motion were integrated using force and moments as input and joint angles (kinematics) as output. However, the joint angle values (prescribed motion) were constrained to follow the input motion (i.e., joint angles computed during motion analysis) with high precision (0.01%). At the end of each time interval, joint moments required to generate the prescribed motion at that instant of time were computed and applied to the model (Dynamics Pipeline, Musculographics Inc., Santa Rosa, CA, USA).

Muscle forces were computed using a static optimization algorithm (Anderson and Pandy 2001) and Hill-type muscle models. In the static optimization, the objective function was to minimize the sum of squared muscle stresses, and the constraint was to equalize the joint moments computed using muscle forces with those computed through inverse dynamics analysis (Dynamics Pipeline, Musculographics Inc., Santa Rosa, CA, USA).

A second inverse dynamics analysis was performed with the computed muscle forces and external forces (ground reaction forces, inertial forces and gravity) as input, in order to compute the resultant hip contact force (HCF).

#### Muscle groups contribution to resultant hip contact force

To study the effect of different muscle groups on femoral head growth tendency, the resultant HCF was decomposed into contributions from individual muscle groups. The GRF was first decomposed into a number of components caused by muscle groups’ forces, using pseudo-inverse acceleration analysis (Lin et al. 2011; Dorn et al. 2012a, b).

**Fig. 1:**
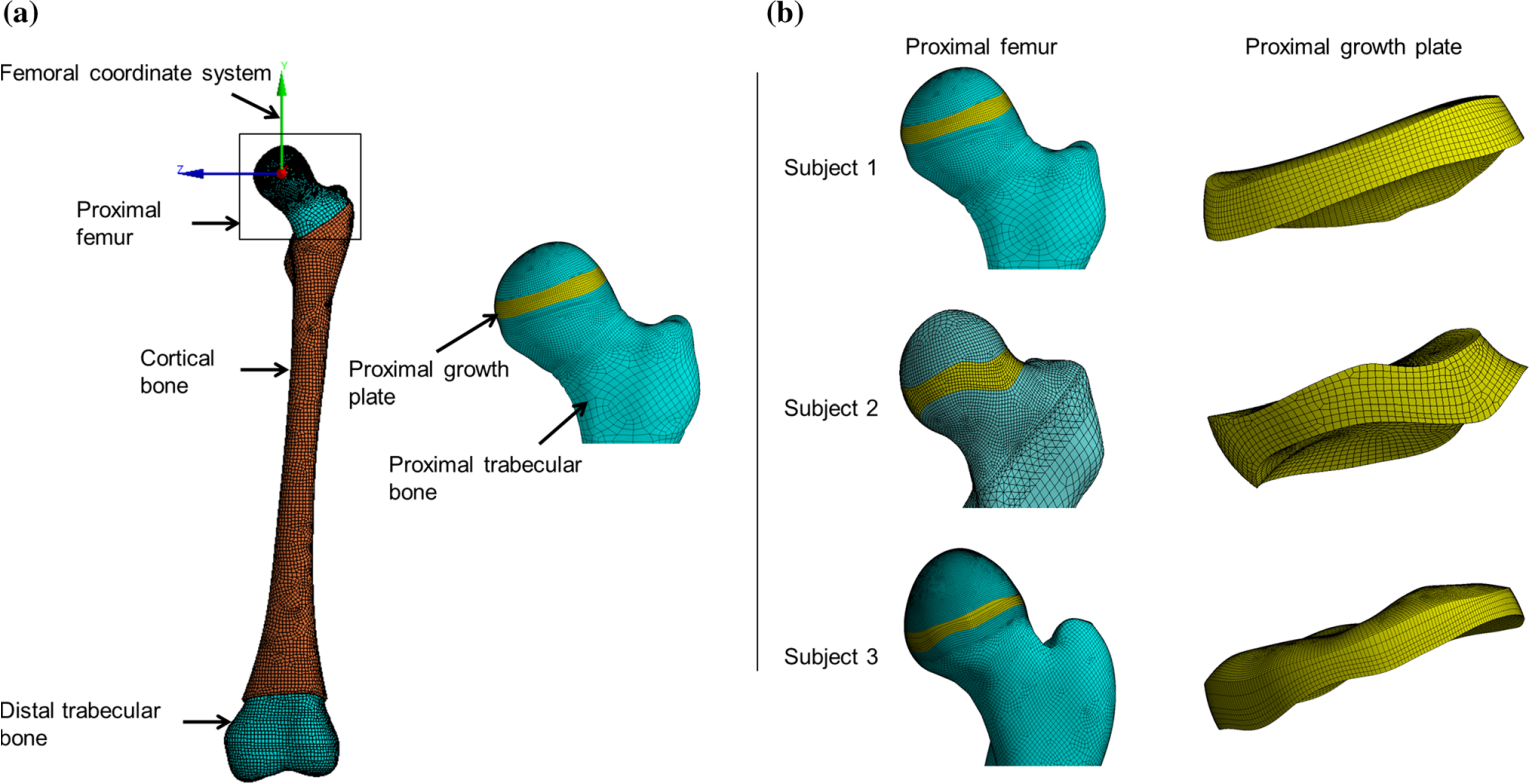
**a** Subject-specific femur model with its components. **b** Proximal femur and growth plate model (*frontal view*)

Individual muscle group’s forces (computed in Sect. 2.3.2), together with their contribution to the GRF (computed in Sect. 2.3.3), were then applied to the modified-scaled model. Inverse dynamics analysis was performed to compute the contribution of that individual muscle group to the overall HCF.

As such, HCF components were computed in order to investigate the individual effects of following muscle groups:

- Full load: All muscles, from Sect. 2.3.2. Full load indicates the resultant HCF
- Hip flexor load: Pectineus, iliacus, psoas, tensor fasciae latae, sartorius, and rectus femoris
- Hip extensor load: Gluteus maximus, biceps femoris, semimembranosus, and semitendinosus
- Hip adductor load: Adductor longus, adductor brevis, adductor magnus, pectineus, quadratus femoris, and gracilis
- Hip abductor load: Gluteus medius, gluteus minimus, gemellus, piriformis, tensor fasciae latae
- Knee extensor load: Rectus femoris, vastus medialis, vastus lateralis, and vastus intermedius

The HCF was computed at the hip joint center (Dynamics Pipeline, Musculographics Inc., Santa Rosa, CA, USA) and described in the femur coordinate system recommended by the International Society of Biomechanics (Wu et al. 2002).

Further, the relative contribution of each considered muscle group to the total HCF was determined as the ratio of area of the HCF plot of that particular muscle group’s load case to the full load case.

### Finite element analysis

#### Mesh and material

3D solid models were generated from the femur surface models. Each femur model consisted of proximal and distal trabecular bone, bone marrow, cortical bone, a proximal growth plate located at the femoral head and transition zones immediately above ($\sim$6  $\hbox {mm}$) and below ($\sim$12 $\hbox {mm}$) the growth plate (Fig. 1). The transition zones were defined for smooth transition of the material properties between the trabecular bone and the growth plate.

To avoid volumetric mesh locking (Puso and Solberg 2006) and to create the rows of nicely aligned elements for growth simulation, the growth plate was meshed with hexahedral elements (20 nodes). Also, to reduce the total number of elements, a hexahedral *dominant* mesh was created for the remaining femur, i.e., locations with regular shapes were meshed with hexahedral elements and any locations with sharp changes in the curvature or complex shapes were meshed mostly with tetrahedral elements (10 nodes). The growth plate of subject 1, 2 and 3 were meshed with element side size of 0.5, 0.8 and 0.5 mm, respectively. The element size for growth plates were determined from the convergence study for peak HCF load (full load case) and with the criterion that relative change in osteogenic index should be less than 5% (ANSYS Inc., Canonsburg, PA). The transition zone volumes were also meshed with the same size element as the growth plate. This meshing generated 4–5 rows ($\sim$minimum thickness of the growth plate/ element size) of elements for growth plate volumes. The element side sizes for the remaining part of the femur model were varied between 1 mm (near transition zone and critical geometry) to 3 mm (predominantly for bone marrow, distal trabecular bone and cortical bone).

A linear elastic, isotropic and homogeneous material model was used for all parts of the femur model. The moduli of elasticity for cortical bone, bone marrow, proximal trabecular bone, and distal trabecular bone were considered as 20 GPa, 1, 58, and 2942 MPa, respectively (Goldstein 1987; Carriero et al. 2011). The growth plate was modeled with a modulus of elasticity 6 MPa and Poisson’s ratio of 0.49 (Shefelbine et al. 2002). The Poisson’s ratio value for all other materials was considered as 0.3 (Carriero et al. 2011). The modulus of elasticity of the transition zones was linearly varied from the growth plate to that of trabecular bone.

#### Loading and boundary condition

**Fig. 2:**
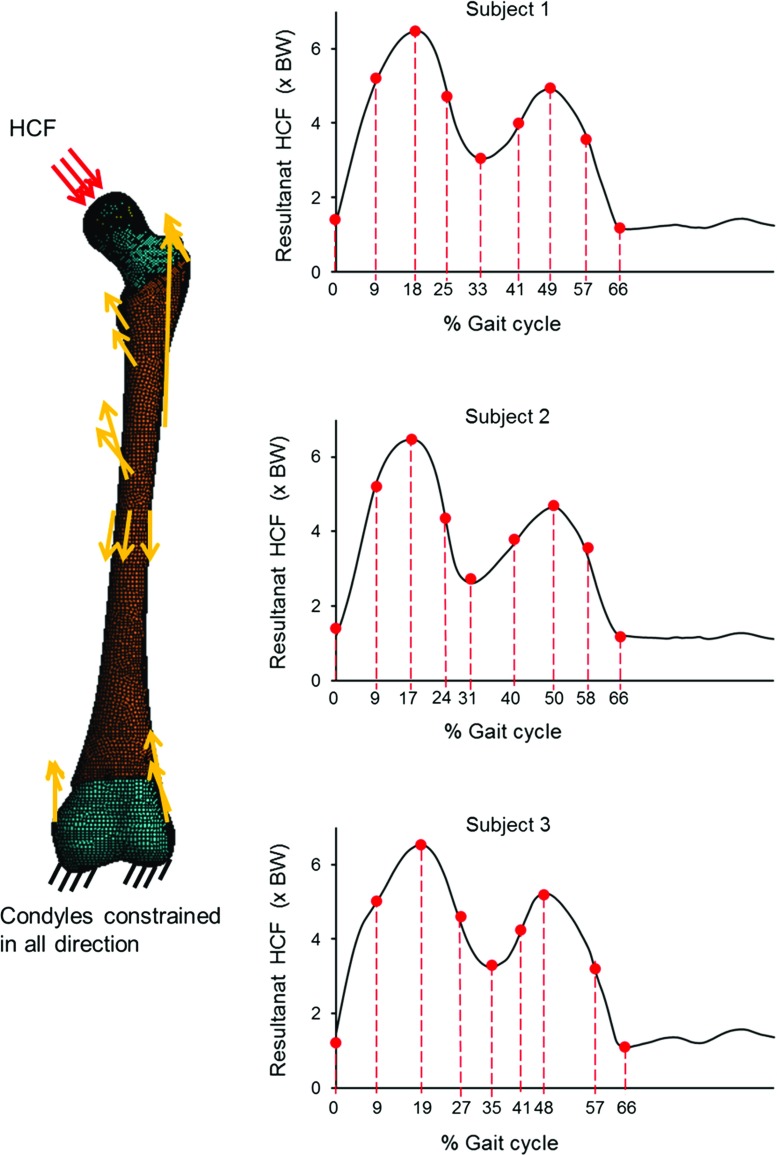
Finite element model and boundary condition. The *yellow arrows* represent muscle forces, not scaled to magnitude. The applied muscle force values are provided in the ESM_1. The resultant HCF *graph* for each subject is shown on the *right side*, with the *red dots* indicating the load instances applied in FE simulation

In the finite element (FE) analysis, one gait cycle was represented by nine sequential load instances (shown in Fig 2); five load instances corresponded to initial contact, first peak, valley and second peak of the resultant HCF, and foot off, and the remaining four load instances were midpoints between those five.

Each muscle force were applied as a concentrated force at a node at the approximated centroid of its attachment site, in the tendon direction. The muscle attachment locations with respect to the femoral coordinate system were found from the musculoskeletal models (developed in Sect. 2.3.1). The muscle forces were applied for the muscles according to the load cases (described in Sect. 2.3.3). To avoid unrealistic stress concentrations around the growth plate region, the HCF was applied as a constant distributed load over the nodes of the femoral head surface (an area of approximately $20\, \hbox {mm}^{2})$ nearest the HCF’s line of action. A preliminary numerical analysis showed that the stresses on the proximal femoral growth plate are mainly due to the HCF (ESM_1). However, muscle forces help to achieve a realistic deformation and restrict tumbling of the femur model in numerical simulation. To restrict rigid body motion of the femur in the numerical analysis, the femoral condyles were constrained in all directions (Taylor et al. 1996; Polgár et al. 2003; Fritz et al. 2009) (Fig. 2). While this constraint may not accurately represent the function of the passive soft tissue structures at the knee joint (Phillips 2009), it has little effect on proximal growth plate stresses (Polgár et al. 2003).

### Growth analysis

#### Growth rate computation

The specific growth rate (${\dot{\varepsilon }})$ was considered as the sum of the biological specific growth rate (${\dot{\varepsilon }}_\mathrm{{b}})$ and the osteogenic index ($I_\mathrm{{O}})$ (Eq. 1).

1 $$\begin{aligned} {\dot{\varepsilon }} = {\dot{\varepsilon }}_\mathrm{{b}}+I_\mathrm{{O}} \end{aligned}$$

where

2 $$\begin{aligned} {\dot{\varepsilon }}=\frac{1}{l}\frac{\mathrm{{d}}l}{\mathrm{{d}}t} \end{aligned}$$

*l* : thickness of the growth zone and $\frac{\mathrm{{d}}l}{\mathrm{{d}}t}$ : growth rate (ESM_1)

In this study, the biological specific growth rate was assumed as constant and determined based on the assumption that 67% of the bone growth is due to biological factors (Hall and Herring 1990; Germiller and Goldstein 1997) and 33% is due to mechanical factors, i.e.,:

$$\begin{aligned} {\dot{\varepsilon }}_\mathrm{{b}}=0.67 {\dot{\varepsilon }}\\ I_\mathrm{{o}} =0.33 {\dot{\varepsilon }} \end{aligned}$$

Or,

3 $$\begin{aligned} {\dot{\varepsilon }}_\mathrm{{b}}\approx 2I_\mathrm{{o}} \end{aligned}$$

Eqs. (2) and (3) can be substituted into Eq. (1), yielding:

4 $$\begin{aligned}&\frac{1}{l}\frac{\mathrm{{d}}l}{\mathrm{{d}}t}={\dot{\varepsilon }}_\mathrm{{b}}+\frac{{\dot{\varepsilon }_\mathrm{{b}}}}{2} \end{aligned}$$

5 $$\begin{aligned}&\frac{1}{l}\frac{\mathrm{{d}}l}{\mathrm{{d}}t}=\frac{3}{2}{\dot{\varepsilon }}_\mathrm{{b}} \end{aligned}$$

6 $$\begin{aligned}&{\dot{\varepsilon }}_\mathrm{{b}}=\frac{2}{3l}\frac{\mathrm{{d}}l}{\mathrm{{d}}t} \end{aligned}$$

In other words, the biological specific growth rate (${\dot{\varepsilon }}_\mathrm{b})$ was considered as the 2/3 of the overall specific growth rate ($\frac{1}{l}\frac{\mathrm{{d}}l}{\mathrm{{d}}t})$. The specific growth rate value used in Eq. (6) was the ratio of the approximate growth rate magnitude ($\frac{\mathrm{{d}}l}{\mathrm{{d}}t})$ reported in the literature (Pritchett 1992) to the mean growth plate thickness (*l*) (Table 1). The computed values of the biological specific growth rate (${\dot{\varepsilon }}_\mathrm{b})$ for the three growth plate models are given in the appendix (ESM 1).

The osteogenic index was computed as (Stevens et al. 1999):

7 $$\begin{aligned} I_o =a \cdot \hbox {max}{\mathrm{\upsigma }}_{\mathrm{Si}} +b \cdot \hbox {min}{{\upsigma }}_{\mathrm{Hj}} \qquad i,j=1,\ldots ,9 \end{aligned}$$

where octahedral shear stress (${\upsigma }_\mathrm{S} )$ and hydrostatic stress (${\upsigma }_\mathrm{H} )$ were computed for the elements of the growth plate surface closest to metaphysis, using all three principal stresses $(\upsigma 1, \upsigma 2, \upsigma 3$) as mentioned in Eqs. (8) and (9). The *i* and *j* in Eq. (7) indicate the 9 load instances during the gait cycle.

8 $$\begin{aligned} {\mathrm{{\upsigma }} }_\mathrm{S}= & {} \frac{\sqrt{\left( {{\mathrm{{\upsigma }} }_1 -{\mathrm{{\upsigma }} }_2 } \right) ^{2}+\left( {{\mathrm{{\upsigma }} }_2 -{\mathrm{{\upsigma }} }_3 } \right) ^{2}+\left( {{\mathrm{{\upsigma }} }_3 -{\mathrm{{\upsigma }} }_1 } \right) ^{2}}}{3} \end{aligned}$$

9 $$\begin{aligned} {\mathrm{{\upsigma }} }_\mathrm{H}= & {} \frac{\mathrm{{\upsigma }} _1 +\mathrm{{\upsigma }} _2 +\mathrm{{\upsigma }} _3 }{3} \end{aligned}$$

For the full load (all muscles) case, max and min in Eq. (7) refer to maximum ${\mathrm{{\upsigma }} }_\mathrm{S}$ and minimum ${\mathrm{{\upsigma }} }_\mathrm{H}$ obtained over the nine gait instances during the gait cycle. For muscle groups’ individual loads, $\upsigma _\mathrm{S}$ and ${{{\upsigma }} }_\mathrm{H}$ were determined as stresses during the same load instances in the full load, i.e., *i* and *j* were determined from the HCF resultant, in order to analyze the growth during walking (full load case) as sums of components due to independent muscle groups. Coefficients *a* and *b* determine the relative influence of ${\mathrm{{\upsigma }} }_\mathrm{S}$ and ${\mathrm{{\upsigma }} }_\mathrm{H}$ on $I_\mathrm{{O}}$. Consistent with previous studies, the *b* / *a* ratio was chosen as 0.5 (Carriero et al. 2011; Shefelbine and Carter 2004a, b; Yadav et al. 2016). The magnitude of the constants *a* and *b* were chosen such that the maximum mechanical contribution to the specific growth was about half of the biological contribution (ESM_1).

#### Growth direction computation

The growth direction was chosen as the direction of maximum (absolute magnitude) principal stress occurring over the nine load instances. The growth direction was computed for each element of the distal surface of growth plate (Yadav et al. 2016).

**Fig. 3:**
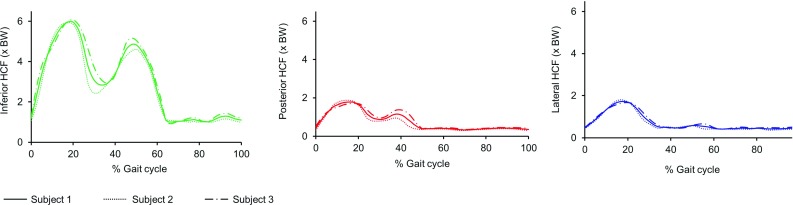
The inferior, posterior and lateral components of the computed hip contact force for three subjects

**Fig. 4:**
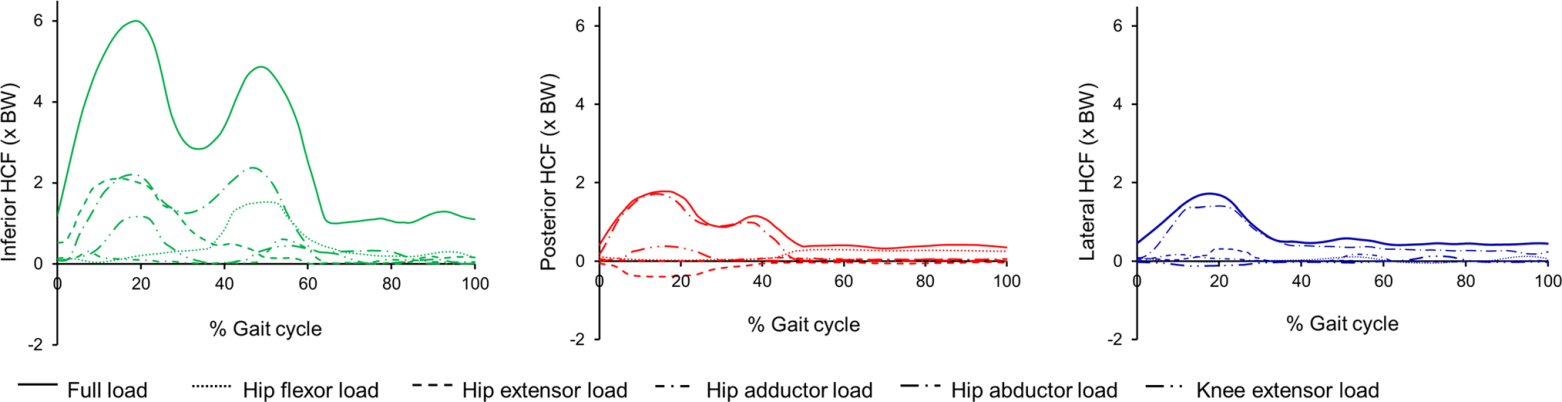
The computed components of different muscle groups to the resultant HCF in a gait cycle for Subject 1. Similar graphs for Subjects 2 and 3 are provided in the ESM_1

The elements of the growth plate surface closest to the metaphysis, which resembles the hypertrophic zone, were “grown” with the magnitude of the specific growth rate and in the principal stress growth direction. To grow the growth plate elements, orthonormal thermal expansion of the elements was used. For each element of the growth region, a new coordinate system was defined, where $x\hbox {-}$, $y\hbox {-}$ and *z*-axes represented the directions of maximum, medium and minimum principal stresses, respectively. Here, maximum, medium and minimum principal stresses were defined based on their numerical value. The coefficient of thermal expansion was defined 1 in the *x*-direction, if the maximum principal stress (absolute magnitude) was highest among all three principal stresses of nine load instances, otherwise in the *z*-direction. The coefficient of thermal expansion in the remaining two directions was defined as zero. The specific growth rate was applied as temperature loading. After the thermal expansion of one row of elements, the nodal coordinates of the complete femur model were updated. The material property of the grown growth plate elements was changed to those of the adjacent transition zone material property, i.e., for further simulations the grown row of elements were considered as part of the transition zone. The stress analysis for all nine load instances was again performed with the updated model and with unchanged HCF and muscle forces. The specific growth rate was again computed, and thermal expansion was again performed for second lower row of elements of the growth plate. These simulations were repeated for four rows of elements of the growth plate, wherein the growth of one row of elements represents the growth for approximately one month. Thus, total growth was simulated for approximately four months. Four rows of elements were chosen to simulate as much growth as possible; more rows of elements were not possible due to the challenge associated with creating regular layers of very small elements, as the growth plate geometry is very irregular.

After growth of four rows of elements of the growth plate, change in NSA and FA was computed for all load cases of all three subjects. The new coordinates of the femoral head center after growth were extracted. The neck shaft axis was redefined with the new coordinates of the femoral head center. The NSA and FA were computed with the new redefined neck shaft axis.

## Results

### Hip contact force

#### Hip contact force for different subjects

The computed HCFs for the three subjects were very similar (Fig. 3). The HCF was found to act in the posterior, inferior and lateral direction over the entire gait cycle for all subjects. The resultant HCF was dominated by the inferior direction component. The inferior, posterior, and lateral direction peak HCF magnitudes were approximately 6, 2, and 2 times body weight, respectively.

**Fig. 5:**
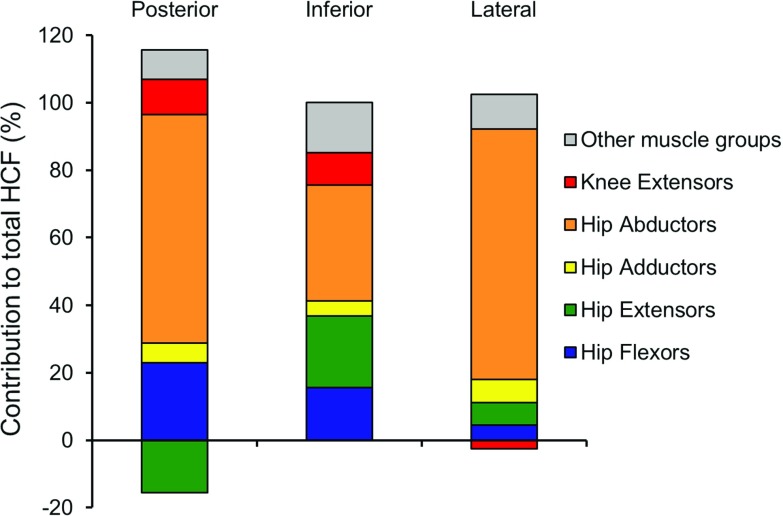
A superposition of muscle groups’ contributions to the resultant HCF (full load case). This indicates that the majority of the HCF is comprised of the 5 included muscle groups. The contributions of all other muscle groups (muscles: extensor digitorum longus, extensor halluces longus, flexor digitorum longus, flexor halluces longus, gastrocnemius, peroneus brevis, peroneus longus, peroneus tertius, soleus, tibialis anterior, tibialis posterior and patellar ligament) together are indicated in *gray*. Their individual influences on HCF can thus be considered negligible

#### Contribution of different muscle groups to resultant hip contact force

The contribution of different muscle groups to the resultant HCF for Subject 1 is shown in Figs. 4 and 5, and for Subjects 2 and 3 in the ESM_1. The directions of HCF components for hip flexors, hip adductors, hip abductors and knee extensors were similar as in the full load case, but the hip extensors produced an HCF component acting in the anterior direction.

The hip abductors contributed most to the resultant HCF in all directions. Hip extensors and knee extensors contributed considerably at the 1st peak of total HCF, and hip flexors, at the 2nd peak of inferior HCF. The combined contributions of the included muscle groups to posterior, inferior and lateral HCF were 92, 85 and 90%, respectively.

**Fig. 6:**
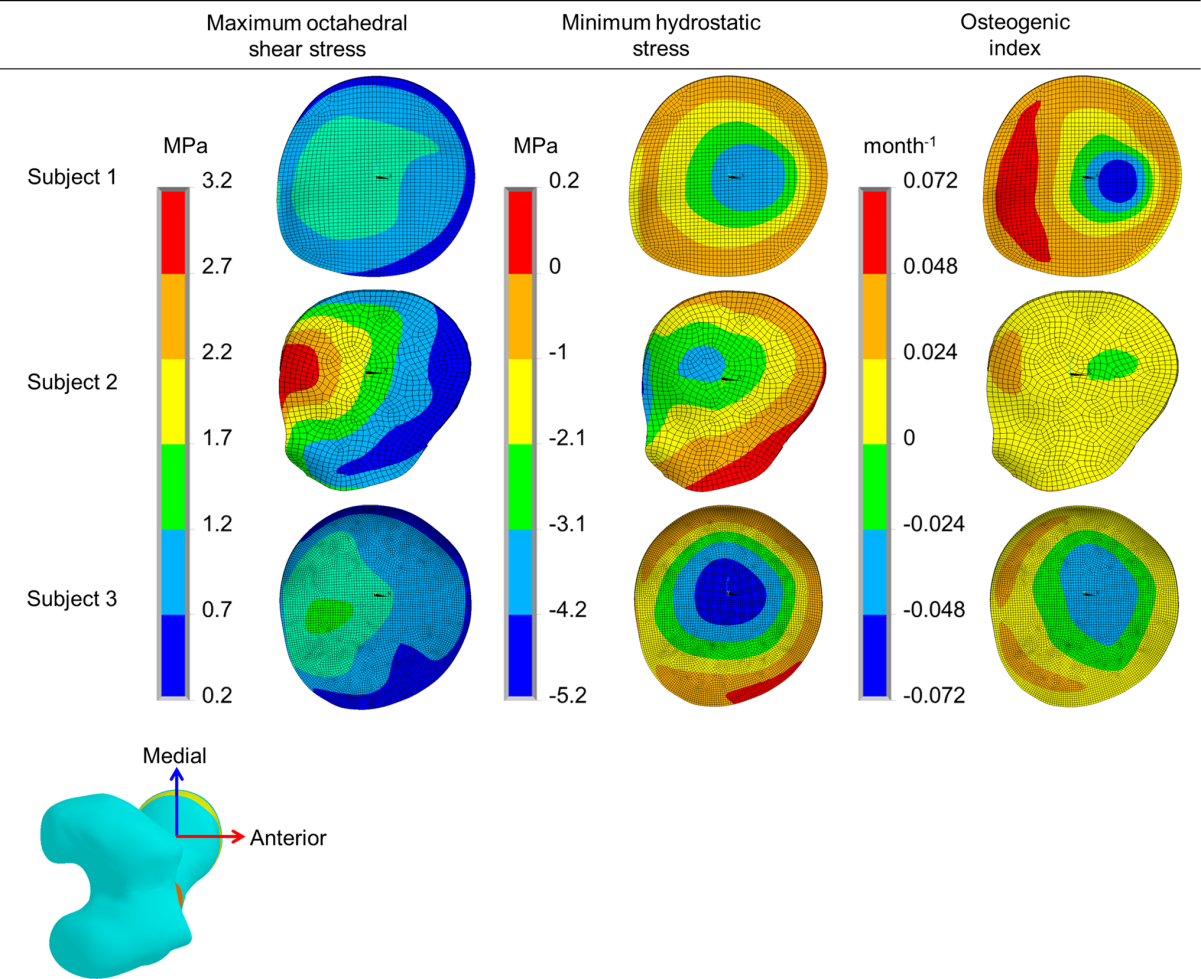
Octahedral shear stress, hydrostatic stress, and osteogenic index on the distal growth plate surface during gait loading for all three subjects

### Growth tendency

#### Overall stresses and growth tendencies

The computed octahedral shear stress, hydrostatic stress and osteogenic index due to gait loading for the three subjects are shown in Fig. 6. Although the patterns and magnitude of overall stresses and osteogenic index were not exactly the same, the locations of maxima and minima were very similar for the three subjects.

**Fig. 7:**
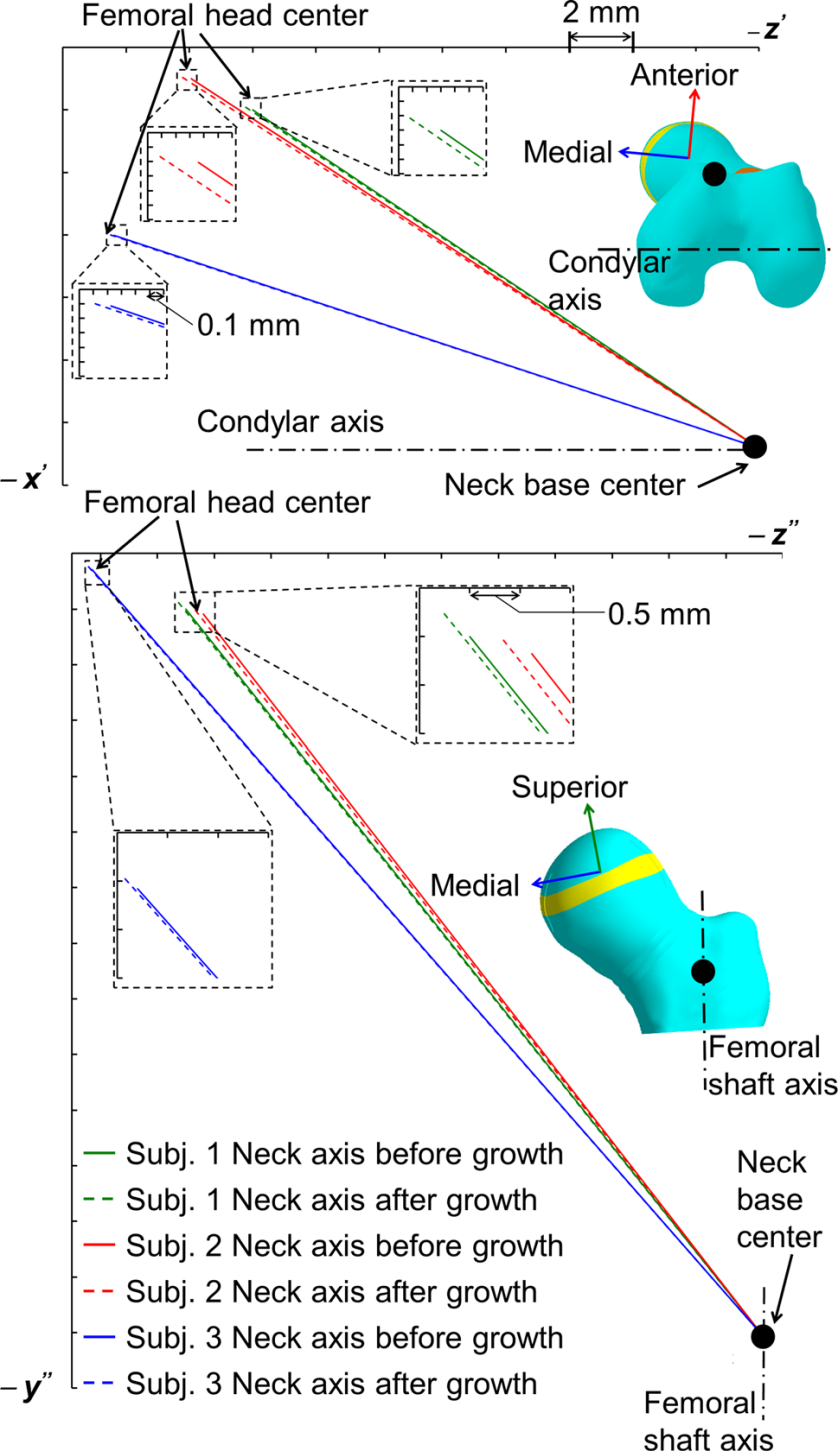
The neck shaft axis orientation (before and after growth) for all three subjects. $z'$ *axis* :  parallel to the projection of the condylar axis onto the anterior-medial plane of the femur coordinate system. $x'$ *axis*: perpendicular to the $z'$ *axis*. $y''$ *axis*: parallel to the projection of the femoral shaft axis onto the superior-medial plane. $z''$ *axis*: perpendicular to the $y''$ *axis*

The maximum octahedral shear stress was found in the posterior region of the growth plate for all subjects. The hydrostatic stress was found to be entirely compressive for Subject 1, but for Subjects 2 and 3, tensile hydrostatic stresses were observed in the anterior-lateral region. The maximum osteogenic index was found in the posterior region, and the minimum, around the center of the growth plate for all subjects. The orientations of neck shaft axis after growth for all three subjects are illustrated in Fig. 7. After growth, the femoral head center was displaced more in the medial direction than in the anterior direction. All three subjects showed a tendency for reduced NSA and FA during growth (Table 2). Subject 3 showed the least reduction in NSA and FA.

#### Growth tendency due to different muscle groups

**Fig. 8:**
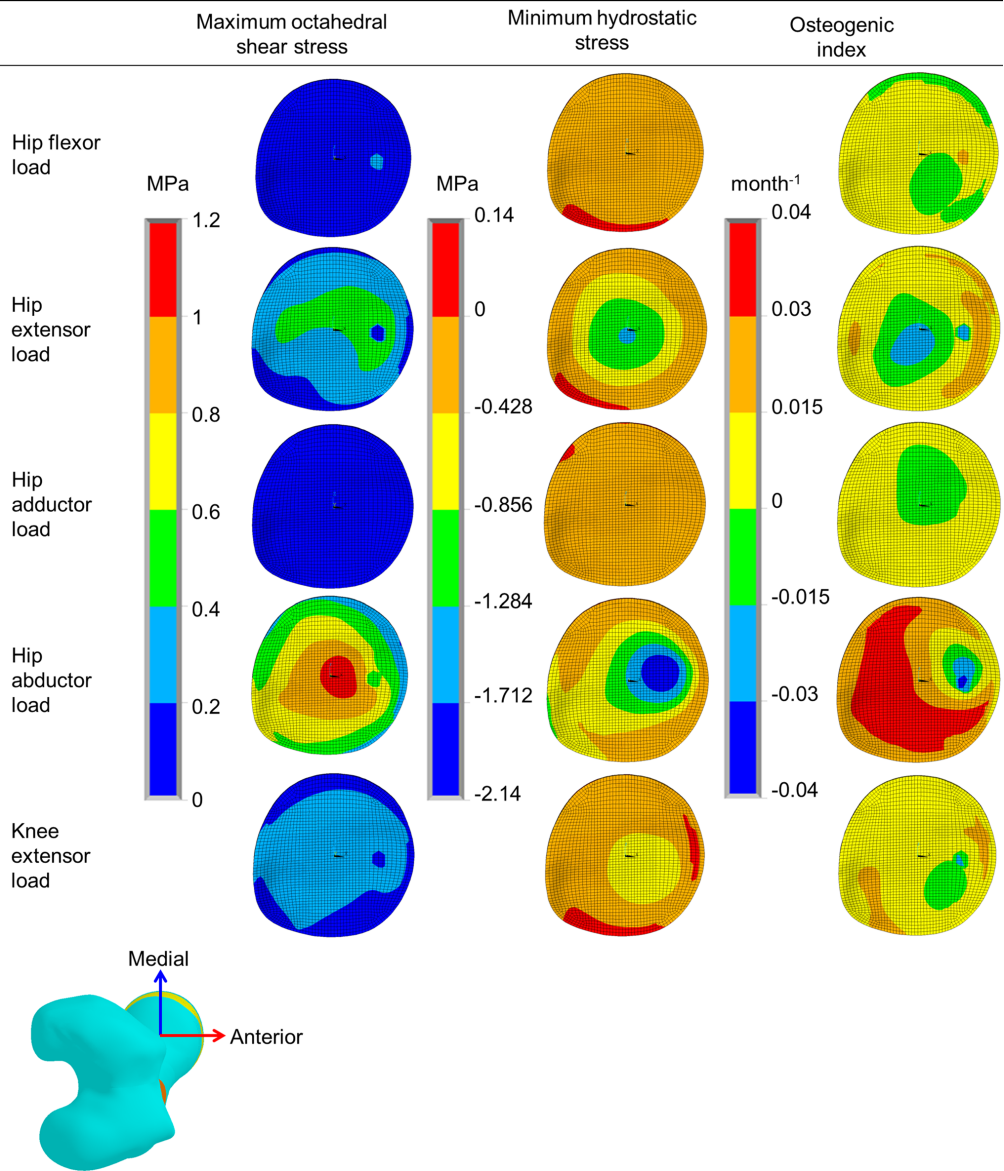
Octahedral shear stress, hydrostatic stress and osteogenic index distribution over the distal surface of the femoral proximal growth plate due to different muscle groups’ forces for Subject 1. The results for Subject 2 and 3 are provided in the ESM_1

**Fig. 9:**
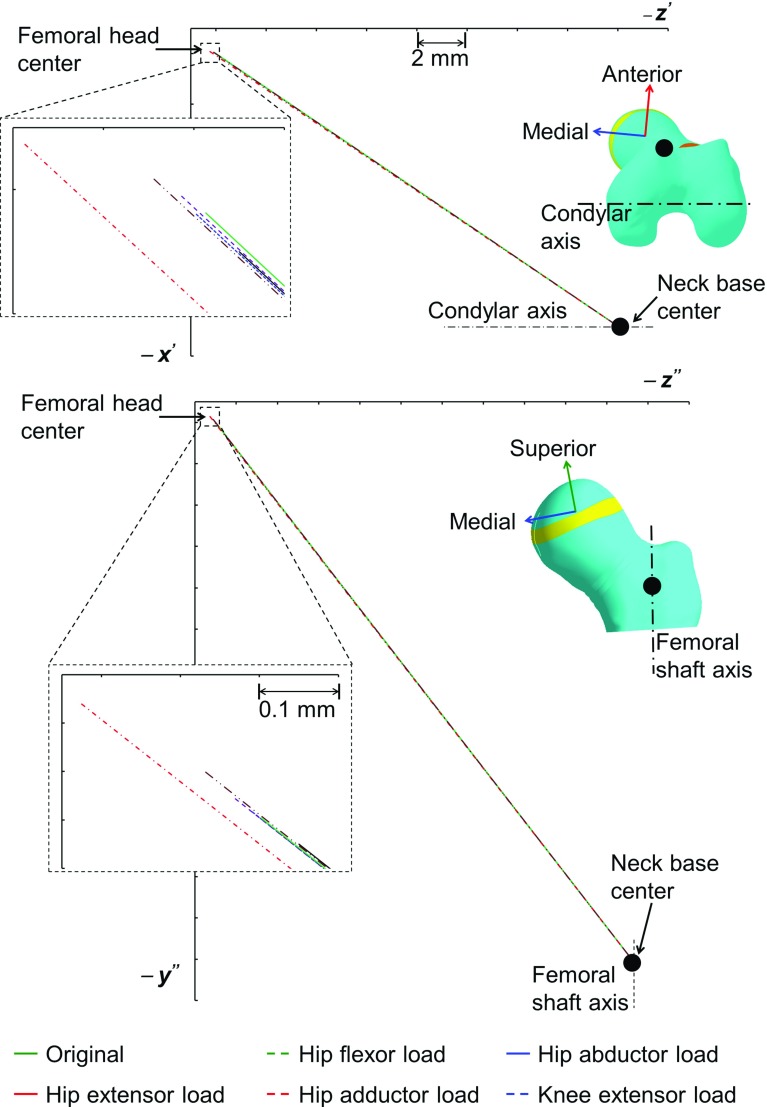
The neck shaft axis orientation in femur coordinate system (original and after growth) due different muscle group loading (for Subject 1). $z'$ axis :  parallel to the projection of the condylar axis onto the anterior-medial plane of the femur coordinate system. $x'$ axis: perpendicular to the $z'$ axis. $y''$ axis: parallel to the projection of the femoral shaft axis onto the superior-medial plane. Axis $z''$: perpendicular to the $y''$ axis. Similar plots for Subjects 2 and 3 are provided in ESM_1.

As the growth tendency was similar for all three subjects, the growth tendency due to different muscle groups is shown only for Subject 1. The stress, osteogenic index and growth tendency results for Subjects 2 and 3 are provided in the ESM_1. The stresses and osteogenic index for individual muscles group are illustrated in Fig. 8. The orientation of neck shaft axis after growth for different muscles groups is shown in Fig. 9, and the changes in NSA due to different muscle groups are reported in Table 3.

Hip flexor load: The overall octahedral shear stress, hydrostatic stress, osteogenic index and femoral head displacement (after growth) magnitudes were lower compared to all other load cases. The hip flexors were found to decrease the NSA and FA.

Hip extensor load: The maximum octahedral shear stress and the maximum compressive hydrostatic stress were around the central region of the growth plate. The osteogenic index was highest in the anterior region and lowest around the center of the growth plate. After growth the femoral head center displacement was highest in the superior direction, followed by the medial and then anterior directions. The hip extensors showed a tendency to decrease the NSA and increase the FA.

Hip adductor load: The octahedral shear stress, compressive hydrostatic stress, and osteogenic index magnitudes were found least compared to all other load cases. Similar to hip extensor load case, the hip adductors resulted the growth that moved the femoral head center primarily in the superior direction followed by medial and then anterior direction. The hip adductors also showed a tendency to reduce the NSA and increase the FA.

Hip abductor load: The octahedral shear stress, compressive hydrostatic stress and osteogenic index magnitudes were highest for this muscle group compared to all other muscle groups. Maximum octahedral shear stress and maximum compressive hydrostatic stress were around the center region of the growth plate. The osteogenic index was higher in the posterior region of the growth plate. The hip abductors resulted in more predicted growth than any other muscle group, with the highest femoral head displacement after growth in the superior direction, followed by the medial and anterior directions. The hip abductors showed a tendency to reduce the NSA and FA.

Knee extensor load: The maximum octahedral shear stress and the maximum compressive hydrostatic stress were found around the center. Higher osteogenic index was observed in the anterior-medial and posterior-lateral regions of the growth plate. Similar to the hip abductors, the knee extensor load induced growth that resulted in femoral head center displacement mostly in the superior-medial direction. The knee extensors showed a tendency to reduce the NSA and FA.

## Discussion

The main finding of this study was that during gait in able-bodied children, hip abductor force contributes more than any other muscle group to the proximal femoral growth development by inducing a reduction of the NSA and FA angles. The value of NSA and FA depends on the position of femoral head center, neck base center and knee joint center (Murphy et al. 1987; Arnold et al. 2001; Zwaag et al. 2015). However, the current study determines only the change in femoral head center position due to the growth at proximal femoral growth plate (located in femoral head region). As the femoral neck base center is located on the bitrochanteric cross section (Arnold et al. 2001), NSA and FA will be affected by the growth at greater and even lesser, trochanteric growth plates. Similarly, the knee joint center will also be affected by growth at distal growth plate. As these three growth plates (greater trochanteric, lesser trochanteric and distal growth plates) were excluded from the present study, the change in NSA and FA simulated in this study should be seen as a part of the overall NSA and FA change.

The growth tendency was considered as a combined effect of both growth rate distribution pattern and growth direction. For example, hip flexors and adductors produced lower osteogenic indices but higher changes in NSA than the knee extensors. Also, regardless of the osteogenic index distribution pattern, each muscle group showed a similar tendency for NSA and FA changes, except hip extensors and hip adductors, which showed a tendency to increase the FA.

**Table 2:** Change in NSA and FA for all three subjects after approximately 4 months

	Change in NSA (${^{\circ }})$	Change in FA (${^{\circ }})$
Subject 1	−0.08	−0.16
Subject 2	−0.15	−0.31
Subject 3	−0.04	−0.05

Bone deformity can develop in response to muscle imbalance. Spastic hip adductors are often considered to be one of the factors that can promote increase in FA during growth (Sutherland et al. 1969; Chong et al. 1978; Gage et al. 2009). In the present study, we confirm with modeling that hip adductors can increase the FA. Our findings also showed reduction in FA due to hip flexors and increase in FA due to hip extensors. Hence, deficient hip flexor force may promote the development of higher FA, which is in agreement with an observation reported in literature that soccer players have shorter hip flexors and higher FA (Chiaia et al. 2009).

As the mechanical regulation of bone growth has been modeled using stress analyses at the growth plate, it is important to include both 3D mechanical load acting on the growth plate and growth plate geometry that changes with age (Guevara et al. 2015) in bone growth modeling. As such, subject-specific femur and growth plate geometries were constructed in the current study. In a previous study, growth was simulated using a generic femur—a scaled average-sized adult male femur—and a simplified growth plate model (Carriero et al. 2011). Their predicted increase in FA during growth may therefore have been a reflection of the already decreased FA in the model morphology. Their simplified growth plate was furthermore located close to the femoral neck region, whereas it is in reality located in the femoral head region. In our previous work (Yadav et al. 2016), we examined the effects of a “simplified” growth plate vs. an MRI-based growth plate, and found different osteogenic distribution patterns. The challenges with subject-specific models lie in the time-consuming construction from medical image data required. In the current study, similar growth tendency was observed for all subject models. It is therefore a reasonable assumption that generic age-dependent *pediatric* femur and growth plate models may suffice for growth studies of able-bodied children. If the goal, however, is to predict growth in a patient population prone to hip deformities, subject-specific femur and growth plate models are recommended. Likewise, whether subject-specific or population-specific gait data is used should probably be based on the heterogeneity of the group and on the accuracy required in the prediction.

Longitudinal growth of long bones at the growth plates is in part mechanically modulated (Stokes 2002); the muscle and joint contact forces can regulate the hip joint morphology in growing children. Carriero et al. (2011) reported the variation in NSA and FA development due to different gait patterns. The current study demonstrates how different muscle groups can influence these parameters. The knee extensors, which do not span the hip, can also contribute to the femur’s growth, as they affect the HCF due to dynamic coupling. The muscles around the foot and ankle, who predominate the ‘Other muscles groups’ in Fig. 5, also contribute to the GRF, and thus to HCF, although their effects, as well as those of the knee extensors are much smaller than that of the muscles that span the hip. To our knowledge, this is the first study to report the influence of different muscle groups on proximal femoral growth tendency.

**Table 3:** Change in NSA and FA due to different muscle groups. The presented data is for Subject 1

	Change in NSA (${^{\circ }})$	Change in FA (${^{\circ }})$
Hip flexor load	-0.010	-0.005
Hip extensor load	-0.011	0.004
Hip adductor load	-0.011	0.015
Hip abductor load	-0.043	-0.179
knee extensor load	-0.002	-0.013

There were several limitations to this study. First, influence of different muscle groups on longitudinal growth was analyzed, but muscle groups can also influence the radial growth of the bone. Radial growth may change the location and orientation of the shaft axis and hence NSA and FA, but will likely do so much more slowly than changes in the growth plate. Another modeling limitation is that in the current study growth was simulated only for growth plate located at the femoral head region, which mainly contributes to femoral neck lengthening (Serrat et al. 2007). However, it has been reported that lateral growth at the greater trochanteric growth plate also affects proximal morphological changes (Siffert 1981) by changing the direction of hip abductor muscles and thus the HCF’s direction and position acting over the femoral head (Heimkes et al. 1997). Also it has been reported that during growth, the greater trochanteric growth plate does not deviate far from the femoral longitudinal axis (Struijs et al. 2011). As such, for more precise prediction of proximal femoral morphological changes, the greater, and even lesser, trochanteric growth plates should be included in future studies. Furthermore, in the current study, predicted growth magnitude is controlled by the constants (biological growth rate, ‘*a*’ and ‘*b*’) used in Eqs. (1) and (7), which were estimated based on the available data in the literature (Hall and Herring 1990; Germiller and Goldstein 1997; Shefelbine and Carter 2004a, b; Carriero et al. 2011; Yadav et al. 2016). A change in their values may affect the specific growth rate magnitude, but will not affect the distribution pattern. The current study focus is more on the direction and pattern of growth, rather than true growth magnitude. More accurate values of the included constants can be estimated by validating the numerical model for a larger population over a long time.

Furthermore, growth was simulated only for loading during gait, whereas growing children perform many other activities daily, such as jumping, running, playing sports, and even sitting and lying, i.e., both static and dynamic loading. Also, children affected with severe bone deformity may be less active, and their bone growth may be predominantly affected by static load. It has been reported that the effect of static compressive load on growth inhibition is higher compared to that of dynamic/intermittent compressive load (Benoit et al. 2016). Therefore, to estimate bone growth accurately, growth simulation should ideally reflect a subject’s daily routine load profile. Our analysis intentionally focuses on gait, as gait is a frequent target and outcome measure for clinical interventions in persons with motor disorders. Also, it is currently not possible to validate the computed muscle forces from a static optimization (or any) algorithm (Komi et al. 1992; Schuind et al. 1992; Finni et al. 1998; Fleming and Beynnon 2004; Ravary et al. 2004; Dennerlein 2005). Computed muscle forces from other algorithms may change the pattern of their contributions to the HCF. Different analytical techniques have been developed to estimate muscle forces (Fleming and Beynnon 2004), but static optimization is still the most commonly used method due to its robustness and efficiency compared to other analytical methods (Anderson and Pandy 2001). Also it has furthermore been reported in the literature that different optimization algorithms result in similar muscle force patterns in gait (Lin et al. 2012).

Due to the time-consuming process of creating subject-specific finite element models and analyses, our small sample size does not make it possible to statistically verify our findings. A thorough analysis of how different muscle groups influence the proximal femoral growth development warrants a study with a larger population of able-bodied children. For such a study, we speculate that growth tendencies due to muscle group action will also be sensitive to subjects’ ages, as the growth plate may undergo geometric changes during growth. Furthermore, a planned continuation of this study is to validate growth tendencies with a second MRI after a two year time period.

In conclusion, results from the subject-specific model developed in this study indicate that different muscle groups influence the osteogenic index magnitude and distribution pattern in different manners during gait. All muscle groups showed similar tendencies to influence proximal femoral growth except hip extensors and hip adductors. Normal gait load, which is a combined effect of all individual muscle group’s loads, induces a reduction in both the NSA and the FA in able-bodied children. Understanding growth tendency and its sensitivity to different muscle groups can help in better clinical treatment development for children with affected gait and muscle activations.

## Electronic supplementary material

Below is the link to the electronic supplementary material.

**Electronic supplementary material (ESM) caption** ESM_1: Sensitivity of HCF and MF on growth plate stress. Magnitude of biological specific growth rate, coefficients *a* and *b*. The contribution of different muscle groups to resultant HCF, growth plate stress, osteogenic index and growth tendency for subject 2 and 3. The muscle force magnitudes considered in FE simulations. (pdf 1,483 KB)
