# Supplementary material for: Influence of muscle groups’ activation on proximal femoral growth tendency
Source: Biomech Model Mechanobiol. 2017 Jun 22;16(6):1869–83. doi: 10.1007/s10237-017-0925-3 (PMC5671539; doi:10.1007/s10237-017-0925-3)
Supplement: Supplementary file 1 — Electronic supplementary material (ESM) caption ESM_1: Sensitivity of HCF and MF on growth plate stress. Magnitude of biological specific growth rate, coefficients a and b. The contribution of different muscle groups to resultant HCF, growth plate stress, osteogenic index and growth tendency for subject 2 and 3. The muscle force magnitudes considered in FE simulations. (pdf 1,483 KB) [file 10237_2017_925_MOESM1_ESM.pdf]

Influence of muscle groups' activation on proximal femoral growth tendency

Priti Yadav (1, 2), Sandra J. Shefelbine PhD (4), Eva Pontén MD PhD (3) and Elena M. Gutierrez-Farewik PhD (1, 2, 3,*)

1. KTH Engineering Sciences, Mechanics, Royal Institute of Technology, Stockholm, Sweden
2. KTH BioMEx Center, Royal Institute of Technology, Stockholm, Sweden
3. Department of Women's & Children's Health, Karolinska Institutet, Pediatric Orthopaedic Surgery, Stockholm, Sweden
4. Department of Mechanical and Industrial Engineering, Northeastern University, Boston, USA

* Corresponding author:

Department of Mechanics, KTH

Osquars Backe 18

10044 Stockholm, Sweden

Tel.: +46 8 790 7719

Email: lanie@mech.kth.se

Stress tensor sensitivity to applied forces

To understand the influence of hip contact force and muscle force on the stress profile of growth plate surface, normal and shear stresses (in femur coordinate system) were computed for the following load cases

1. Both HCF and all muscle forces were applied
2. Only HCF was applied
3. Only muscle forces were applied

In this analysis, load applied to the femur model in finite element analysis correspond to peak resultant HCF (instance 3) of full load case. The considered femur model is of subject 1. The result of this study is presented in Fig. S1 and Fig S2

The result of this study suggested that the stress tensor profile at growth plate surface is mostly due to the HCF.

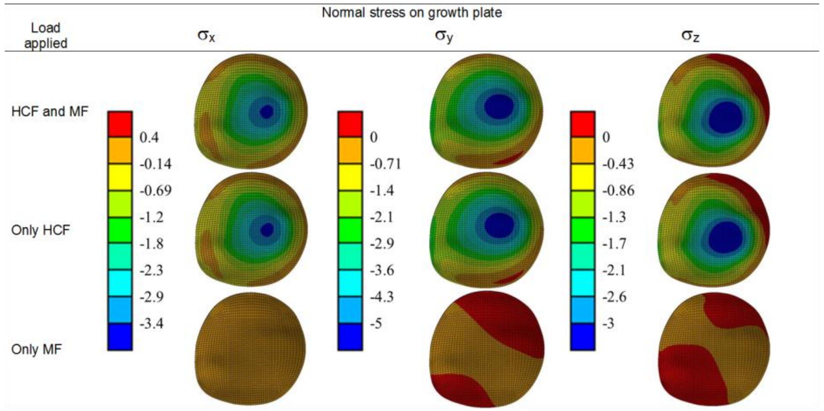

Figure S1. Normal (σ) (MPa) on the distal surface of the growth plate computed with respect to the femur coordinate system. For each row in the figure, the same stress scale was used.

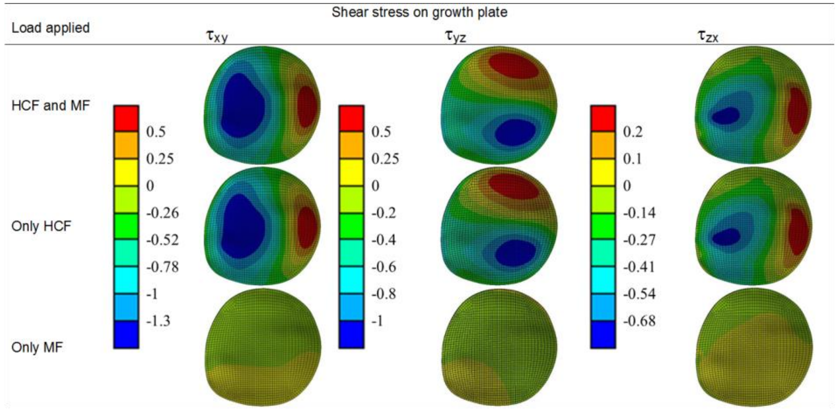

Figure S2. Shear stress (τ) (MPa) on the distal surface of the growth plate computed with respect to the femur coordinate system. For each row in the figure, the same stress scale was used.

Value of constant considered in computation of specific growth rate

Table S1 Biological specific growth rate and coefficients *a* and *b.* The growth rate magnitude was taken from the literature (Pritchett, 1992).

	Gender	Growth rate $\left(\frac{dl}{dt}\right)$ (mm/month)	Biological specific growth rate $\left(\frac{2}{3l}\frac{dl}{dt}\right)$ (month^{-1})	a (month^{-1}Mpa^{-1})	b (month^{-1}Mpa^{-1})
Subject 1	Female	0.89	0.14	0.06	0.03
Subject 2	Male	0.75	0.08	0.0186	0.0093
Subject 3	Female	0.50	0.09	0.035	0.0175

Contribution of different muscle groups to resultant HCF

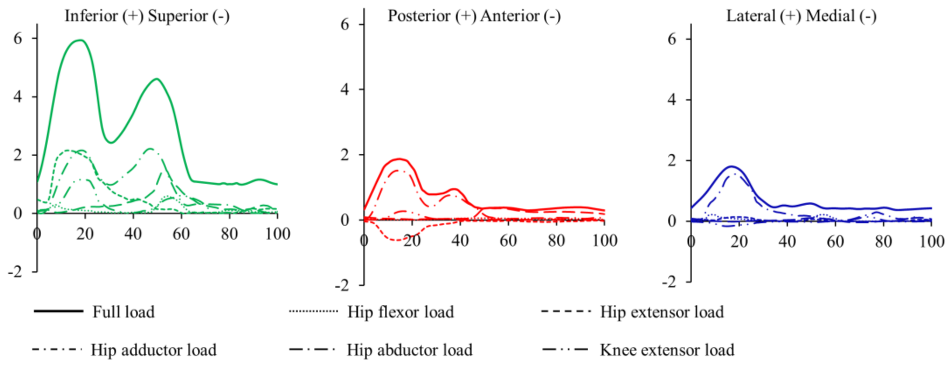

Figure S3 The computed components of different muscle groups to the resultant HCF for subject 2. In the graph HCF (x BW) is ordinate and percentage of gait is the abscissa.

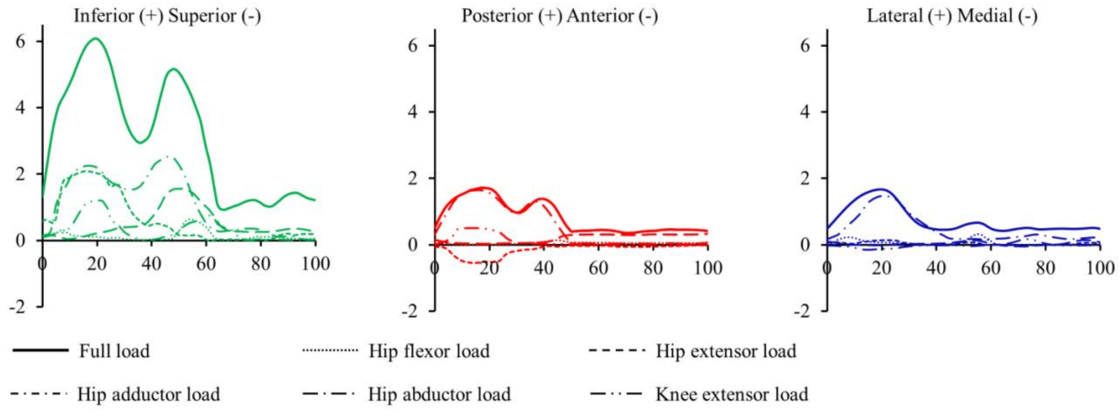

Figure S4 The computed components of different muscle groups to the resultant HCF for subject 3. In the graph HCF (x BW) is ordinate and percentage of gait is the abscissa.

Stresses, osteogenic index and growth tendecy due to different muscle groups (for subject 2 and 3)

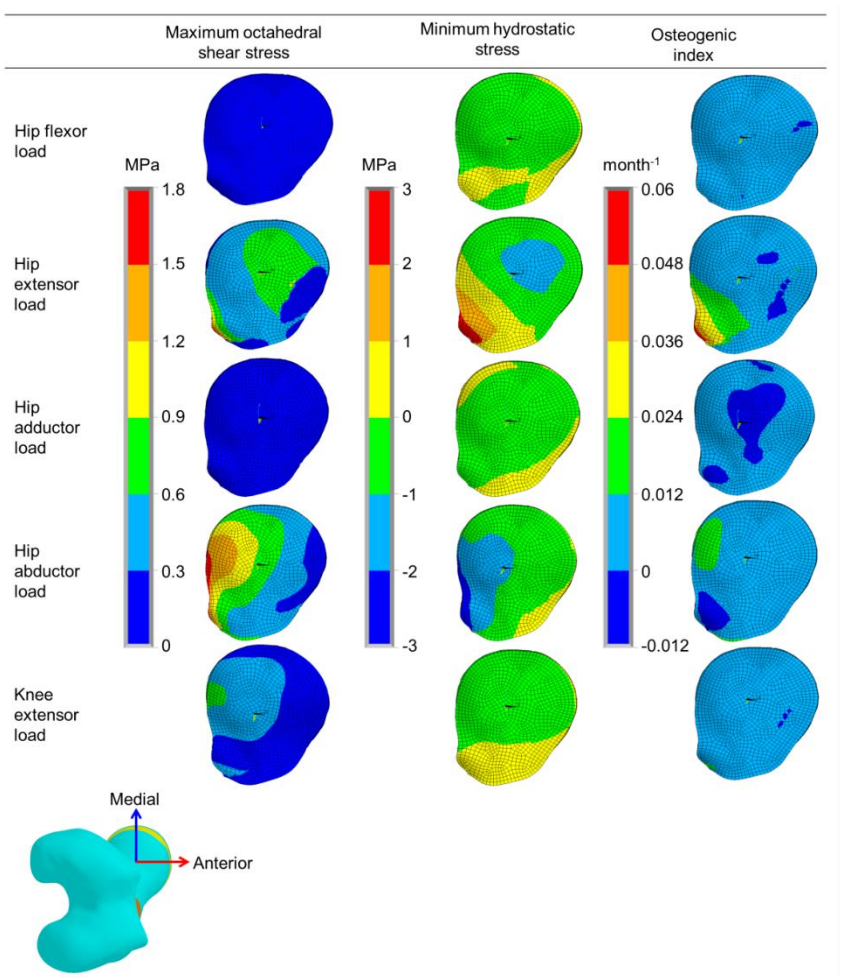

Figure S5 Maximum octahedral shear stress, minimum hydrostatic stress and osteogenic index due to different muscle groups for subject 2.

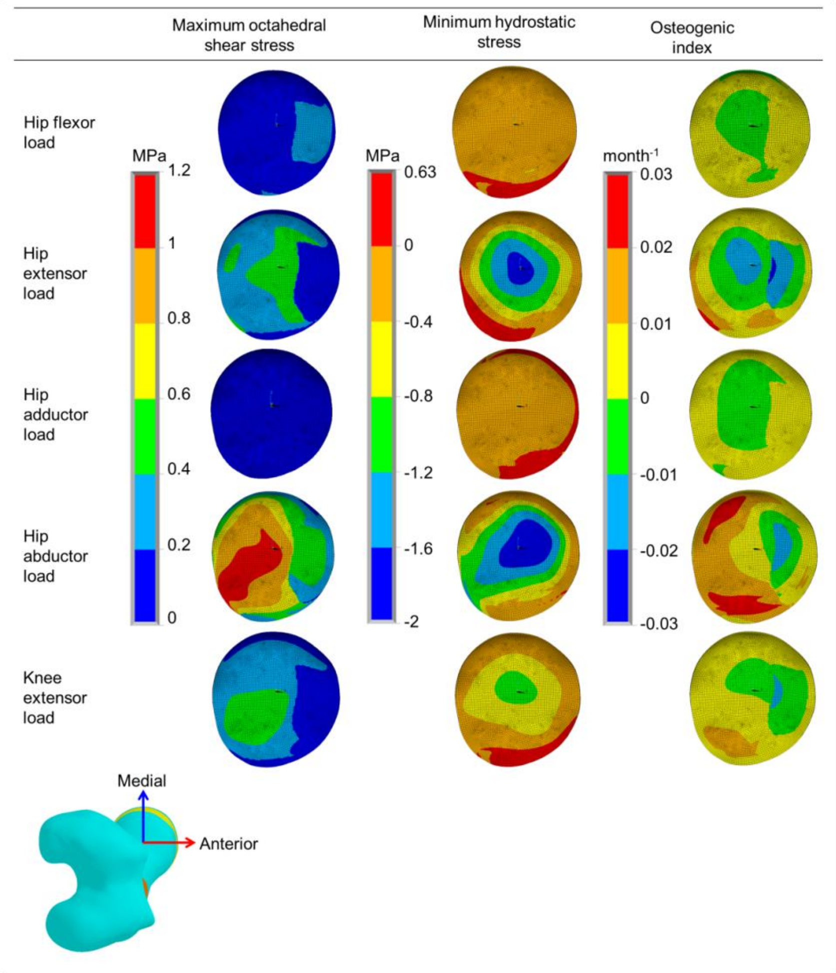

Figure S6 Maximum octahedral shear stress, minimum hydrostatic stress and osteogenic index due to different muscle groups for subject 3.

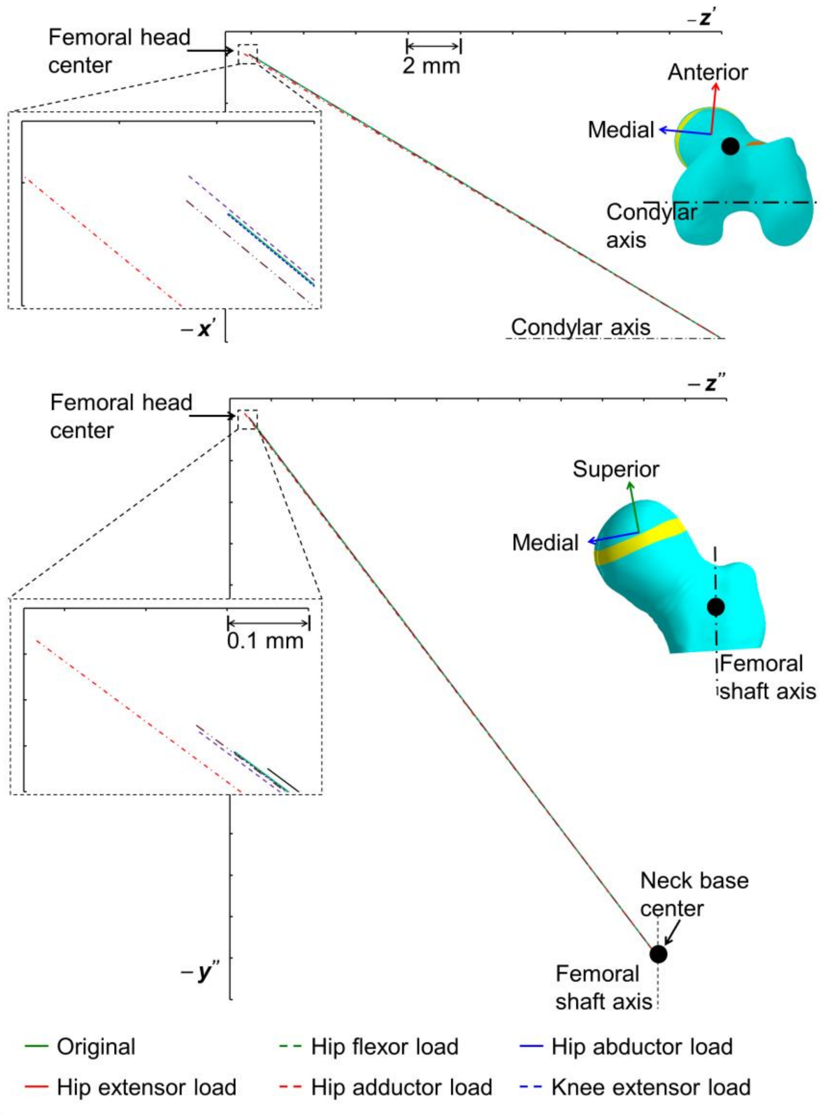

Figure S7 Orientation of the neck shaft axis for subject 2.

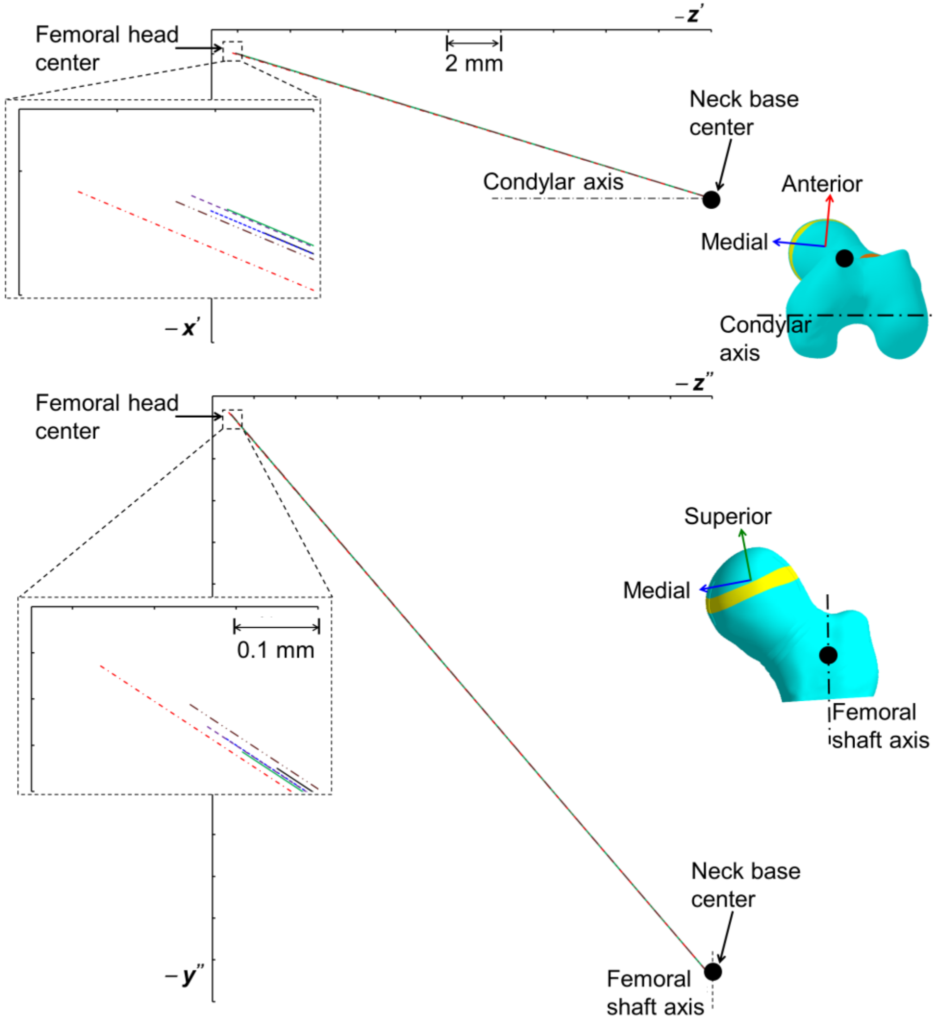

Figure S8 Orientation of the neck shaft axis for subject 3.

Table S2. Change in NSA and FA due to different muscle groups. The presented data is for Subject 2.

	Change in NSA (°)	Change in FA (°)
Hip flexor load	-0.020	-0.003
Hip extensor load	-0.037	0.008
Hip adductor load	-0.019	0.0001
Hip abductor load	-0.022	-0.239
knee extensor load	-0.009	-0.040

Table S3. Change in NSA and FA due to different muscle groups. The presented data is for Subject 3.

	Change in NSA (°)	Change in FA (°)
Hip flexor load	-0.009	-0.001

Hip extensor load	-0.011	0.014
Hip adductor load	-0.017	0.017
Hip abductor load	-0.022	-0.075
knee extensor load	-0.002	-0.012

<u>Muscle forces:</u> The muscle force data used in presented finite element analysis is presented in the Table S4-S12.

Table S4: Subject 1 - Anterior (+) Posterior (-) component of the muscle forces (Unit: N)

Load Instance	1	2	3	4	5	6	7	8	9
percent	0	9	18	25	33	41	49	57	66
Gluteus Maximus Anterior	1.16E+00	3.85E+01	2.64E+01	2.62E+00	2.18E-01	-3.00E-01	-2.63E-02	-1.83E-02	4.50E-02
Gluteus Maximus Middle	1.75E-01	3.34E+00	-6.23E+00	-2.94E+00	-1.21E+00	-4.74E-02	-1.06E-02	-2.23E-02	-3.27E-02
Gluteus Maximus Posterior	-4.51E-01	-4.93E+00	-2.28E-02	-1.48E-02	-1.43E-02	-1.37E-02	-5.68E-03	-1.16E-02	-2.13E-01
Gluteus Medius Anterior	9.20E+00	3.65E+01	1.44E+02	1.01E+02	5.92E+01	3.74E+01	3.91E+01	1.46E+01	4.07E-02
Gluteus Medius Middle	7.49E+00	1.56E+01	3.44E+01	2.34E+01	1.10E+01	4.16E+00	9.10E-01	2.93E+00	4.91E-01
Gluteus Medius	7.22E+00	1.79E+01	1.37E+01	3.69E+00	-8.60E-01	-3.83E+00	-3.62E+00	-1.44E+00	1.47E+00

Posterior									
Gluteus Minimus Anterior	1.83E+00	2.58E+00	1.23E+01	1.12E+01	7.13E+00	4.50E+00	5.58E+00	3.43E+00	2.90E-01
Gluteus Minimus Middle	2.18E+00	2.50E+00	1.07E+01	8.10E+00	4.71E+00	2.84E+00	2.33E+00	1.95E+00	3.73E-01
Gluteus Minimus Posterior	2.92E+00	2.55E+00	8.81E+00	4.13E+00	1.82E+00	4.62E-01	-4.67E-01	3.37E-01	4.50E-01
Adductor Longus	-2.81E-02	-8.65E-03	3.80E-04	1.11E-03	2.21E-03	5.04E-03	4.14E-03	-4.07E-03	1.16E+00
Adductor Magnus Anterior	-2.49E-01	-3.49E-01	-6.68E-03	-3.50E-03	-2.59E-03	-2.09E-05	1.96E-03	-1.47E-03	-8.64E-01
Adductor Magnus Middle	-1.82E-01	-4.54E-01	-6.75E-03	-3.49E-03	-3.31E-03	-2.78E-03	-6.62E-04	-3.83E-03	-4.83E-01
Adductor Magnus Posterior	-1.45E-01	-1.01E+00	-9.82E-03	-4.45E-03	-3.58E-03	-1.93E-03	-1.56E-04	-1.47E-03	-1.30E-02
Adductor Brevis	-1.63E-01	-1.57E-01	-3.33E-03	-1.41E-03	-4.82E-04	2.06E-03	2.85E-03	2.09E-03	-2.55E-01
Iliacus	2.49E+00	9.17E-02	2.91E-01	1.53E+01	2.44E+01	5.64E+01	9.85E+01	7.39E+01	2.46E+01
Pectineus	1.93E-01	1.10E-01	1.17E-02	9.43E-03	1.50E-02	5.38E-02	2.79E-02	1.48E-01	6.50E-01
Piriformis	6.75E-03	-1.87E-01	-4.00E+00	-2.23E+00	-1.33E+00	-2.57E+00	-3.04E-01	-8.47E-01	-1.60E+00
Psoas	2.69E+00	8.73E-02	1.74E+00	2.00E+01	2.80E+01	6.67E+01	1.16E+02	8.37E+01	2.79E+01
Quadratus Femoris	-9.84E-01	-2.53E-01	-1.64E-02	-7.66E-03	-6.06E-03	-2.62E-03	1.99E-03	-5.04E-03	-3.81E+00
Gemelleus	-8.57E-01	-3.00E-01	-8.89E-02	-2.86E-02	-2.72E-02	-7.17E-02	-1.31E-02	-7.50E-02	-8.08E-01
Sartorius	-2.31E-01	-5.12E-02	-2.69E-01	-3.40E-01	-2.95E-01	-2.67E-01	-7.10E-01	-1.17E+00	-5.85E-01
Biceps Femoris Short Head	-1.62E+00	-6.11E-01	-2.04E+00	1.07E-01	-1.42E+00	-2.44E+00	-9.71E+00	-9.45E+00	-1.22E+01
Gastrocnemius Lateral	-1.05E+00	-1.25E-02	-1.73E-02	-1.28E-01	-1.26E+00	-4.96E+00	-1.07E+01	-8.20E+00	-5.65E-01
Gastrocnemius Medial	-4.10E+00	-2.18E-02	-2.19E-02	-6.65E-02	-3.02E+00	-2.05E+01	-4.80E+01	-3.66E+01	-8.89E-01
Tensor Fascia Latae	-4.63E-01	-4.54E-01	-7.51E+00	-5.81E+00	-2.75E+00	-1.51E+00	-3.68E+00	-4.34E+00	-1.00E+00

Vastus Intermedius	1.18E-02	5.29E+00	8.10E+00	4.01E+00	5.56E-01	7.75E-03	4.24E-03	6.95E-03	4.26E-02
Vastus Lateralis	2.48E-02	1.57E+01	2.62E+01	1.40E+01	1.12E+00	1.73E-02	9.28E-03	1.39E-02	8.37E-02
Vastus Medialis	1.78E-02	4.98E+00	8.58E+00	4.70E+00	5.90E-01	1.17E-02	5.82E-03	8.25E-03	3.83E-02

Table S5: Subject 1 - Superior (+) Inferior (-) component of the muscle forces (Unit: N)

Load Instance	1	2	3	4	5	6	7	8	9
percent	0	9	18	25	33	41	49	57	66
Gluteus Maximus Anterior	2.20E+00	8.64E+01	9.03E+01	1.76E+01	6.62E+00	3.36E+00	1.53E-01	3.11E-01	2.74E-01
Gluteus Maximus Middle	2.30E+00	1.04E+02	1.02E+02	1.92E+01	5.26E+00	1.49E-01	2.77E-02	7.37E-02	2.10E-01
Gluteus Maximus Posterior	8.71E-01	8.62E+00	9.26E-02	1.83E-02	1.61E-02	1.51E-02	6.98E-03	1.45E-02	3.13E-01
Gluteus Medius Anterior	5.08E+00	2.43E+01	1.36E+02	1.31E+02	9.98E+01	8.88E+01	1.28E+02	2.99E+01	4.75E-02
Gluteus Medius Middle	7.52E+00	1.84E+01	5.69E+01	5.83E+01	4.41E+01	3.92E+01	5.29E+01	2.07E+01	1.16E+00
Gluteus Medius Posterior	1.28E+01	3.76E+01	4.74E+01	3.43E+01	2.19E+01	2.12E+01	1.39E+01	1.08E+01	1.12E+01
Gluteus Minimus Anterior	9.65E-01	1.67E+00	1.14E+01	1.43E+01	1.20E+01	1.08E+01	1.86E+01	7.08E+00	2.78E-01
Gluteus Minimus Middle	1.70E+00	2.30E+00	1.38E+01	1.47E+01	1.20E+01	1.09E+01	1.69E+01	6.78E+00	6.29E-01
Gluteus Minimus Posterior	3.37E+00	3.42E+00	1.69E+01	1.26E+01	9.78E+00	9.84E+00	1.14E+01	3.89E+00	1.28E+00
Adductor Longus	5.82E-01	3.28E-01	1.85E-02	1.66E-02	2.21E-02	3.44E-02	2.19E-02	-3.23E-02	1.53E+01

Adductor Magnus Anterior	4.33E-01	6.04E-01	1.26E-02	8.86E-03	1.11E-02	1.57E-02	1.01E-02	2.77E-02	3.07E+00
Adductor Magnus Middle	5.71E-01	1.46E+00	2.38E-02	1.49E-02	1.81E-02	2.51E-02	1.46E-02	3.10E-02	2.33E+00
Adductor Magnus Posterior	8.88E-01	6.50E+00	7.33E-02	4.25E-02	4.63E-02	4.80E-02	2.23E-02	3.02E-02	1.36E-01
Adductor Brevis	5.54E-01	5.68E-01	1.55E-02	1.19E-02	1.55E-02	2.24E-02	1.42E-02	4.02E-02	3.74E+00
Iliacus	2.16E+00	8.46E-02	3.14E-01	1.94E+01	3.61E+01	9.59E+01	1.87E+02	1.21E+02	3.08E+01
Pectineus	8.06E-01	3.26E-01	2.68E-02	1.90E-02	2.87E-02	9.59E-02	4.67E-02	2.99E-01	1.46E+00
Piriformis	5.52E+00	2.20E+00	1.34E+01	4.09E+00	1.70E+00	2.38E+00	2.41E-01	9.25E-01	3.53E+00
Psoas	2.39E+00	8.01E-02	1.81E+00	2.45E+01	3.93E+01	1.08E+02	2.04E+02	1.34E+02	3.51E+01
Quadratus Femoris	4.66E-01	6.75E-02	-3.36E-03	-6.41E-03	-1.14E-02	-2.59E-02	-1.17E-02	-2.09E-02	-1.60E+00
Gemelleus	3.59E-01	7.90E-02	-3.74E-03	-9.19E-03	-1.53E-02	-6.20E-02	-1.50E-02	-5.29E-02	-1.67E-01
Sartorius	-1.53E+00	-2.02E-01	-1.12E+00	-1.82E+00	-2.05E+00	-2.13E+00	-3.78E+00	-3.20E+00	-1.13E+00
Biceps Femoris Short Head	-1.39E+01	-3.38E+00	-1.19E+01	7.64E-01	-1.26E+01	-2.43E+01	-6.92E+01	-3.86E+01	-3.79E+01
Gastrocnemius Lateral	-4.45E+00	-2.27E-02	-3.45E-02	-3.77E-01	-5.72E+00	-2.89E+01	-3.13E+01	-7.44E+00	-2.05E-01
Gastrocnemius Medial	-1.95E+01	-4.24E-02	-4.70E-02	-2.15E-01	-1.56E+01	-1.41E+02	-1.54E+02	-3.60E+01	-4.05E-01
Tensor Fascia Latae	-1.97E+00	-9.29E-01	-1.66E+01	-1.80E+01	-1.25E+01	-8.63E+00	-1.13E+01	-5.40E+00	-7.11E-01
Vastus Intermedius	-1.09E-01	-5.28E+01	-7.95E+01	-3.75E+01	-3.90E+00	-7.06E-02	-3.96E-02	-8.78E-02	-1.01E+00
Vastus Lateralis	-1.07E-01	-7.85E+01	-1.28E+02	-6.28E+01	-4.75E+00	-7.19E-02	-4.18E-02	-9.04E-02	-9.62E-01
Vastus Medialis	-1.09E-01	-3.61E+01	-6.03E+01	-3.02E+01	-3.59E+00	-6.95E-02	-3.74E-02	-8.45E-02	-1.08E+00

Table S6: Subject 1 - Medial (+) Lateral (-) component of the muscle forces (Unit: N)

Load Instance	1	2	3	4	5	6	7	8	9
percent	0	9	18	25	33	41	49	57	66
Gluteus Maximus Anterior	1.33E-01	8.51E+00	1.49E+01	3.14E+00	1.11E+00	6.12E-01	3.29E-02	1.10E-02	-6.95E-03
Gluteus Maximus Middle	-1.96E-01	-5.31E+00	-1.57E+00	-2.96E-01	-1.36E-01	1.47E-03	2.66E-03	-3.93E-03	-1.90E-02
Gluteus Maximus Posterior	2.05E-01	2.34E+00	1.07E-02	7.19E-03	7.75E-03	1.04E-02	6.58E-03	9.76E-03	1.43E-01
Gluteus Medius Anterior	4.44E-01	2.93E+00	2.27E+01	2.09E+01	1.29E+01	7.23E+00	3.52E+00	-2.12E+00	-5.57E-03
Gluteus Medius Middle	2.74E+00	7.39E+00	2.56E+01	2.57E+01	1.82E+01	1.53E+01	1.97E+01	4.56E+00	1.98E-01
Gluteus Medius Posterior	6.10E+00	2.02E+01	2.96E+01	2.24E+01	1.43E+01	1.46E+01	1.02E+01	4.91E+00	4.14E+00
Gluteus Minimus Anterior	5.79E-01	9.26E-01	5.83E+00	6.51E+00	4.82E+00	3.61E+00	5.04E+00	1.53E+00	5.78E-02
Gluteus Minimus Middle	9.16E-01	1.24E+00	7.35E+00	7.36E+00	5.49E+00	4.51E+00	6.28E+00	1.84E+00	1.61E-01
Gluteus Minimus Posterior	2.00E+00	2.12E+00	1.11E+01	8.05E+00	5.99E+00	5.88E+00	6.55E+00	1.68E+00	4.93E-01
Adductor Longus	2.85E-01	1.60E-01	8.90E-03	7.86E-03	1.03E-02	1.54E-02	9.20E-03	-1.46E-02	7.19E+00
Adductor Magnus Anterior	4.04E-01	6.03E-01	1.41E-02	1.08E-02	1.44E-02	2.08E-02	1.32E-02	3.55E-02	3.62E+00
Adductor Magnus Middle	1.85E-01	4.76E-01	7.78E-03	5.00E-03	6.35E-03	9.29E-03	5.64E-03	1.27E-02	9.26E-01
Adductor Magnus Posterior	2.02E-01	1.47E+00	1.63E-02	9.53E-03	1.07E-02	1.14E-02	5.45E-03	7.86E-03	3.56E-02
Adductor Brevis	4.77E-01	5.12E-01	1.48E-02	1.16E-02	1.51E-02	2.14E-02	1.30E-02	3.68E-02	3.38E+00

Iliacus	-3.66E-01	-1.69E-02	-5.69E-02	-2.85E+00	-4.66E+00	-1.53E+01	-4.07E+01	-2.20E+01	-6.01E+00
Pectineus	6.20E-01	2.96E-01	2.39E-02	1.61E-02	2.32E-02	7.07E-02	3.10E-02	2.14E-01	1.14E+00
Piriformis	3.89E+00	1.76E+00	1.32E+01	4.66E+00	2.18E+00	3.80E+00	4.66E-01	1.11E+00	2.87E+00
Psoas	2.73E-01	4.17E-03	2.61E-02	3.67E-01	4.92E-01	-3.54E+00	-2.07E+01	-6.22E+00	-7.90E-01
Quadratus Femoris	1.14E+00	3.05E-01	2.20E-02	1.38E-02	1.77E-02	3.58E-02	1.71E-02	4.45E-02	9.83E+00
Gemelleus	1.11E+00	3.87E-01	1.12E-01	3.66E-02	3.71E-02	1.17E-01	2.74E-02	1.25E-01	1.16E+00
Sartorius	-4.48E-02	-6.12E-03	-3.38E-02	-5.36E-02	-5.98E-02	-6.20E-02	-1.11E-01	-1.04E-01	-4.17E-02
Biceps Femoris Short Head	-1.52E+00	-3.81E-01	-1.33E+00	8.46E-02	-1.38E+00	-2.66E+00	-7.67E+00	-4.54E+00	-4.84E+00
Gastrocnemius Lateral	3.82E-01	2.48E-03	3.62E-03	3.49E-02	4.85E-01	2.38E+00	2.89E+00	1.22E+00	5.14E-02
Gastrocnemius Medial	2.08E+00	5.62E-03	6.02E-03	2.45E-02	1.65E+00	1.45E+01	1.76E+01	6.89E+00	1.57E-01
Tensor Fascia Latae	2.55E-01	1.22E-01	2.16E+00	2.32E+00	1.62E+00	1.12E+00	1.46E+00	7.66E-01	1.28E-01
Vastus Intermedius	9.96E-03	4.47E+00	6.80E+00	3.34E+00	3.59E-01	6.61E-03	3.52E-03	6.95E-03	7.82E-02
Vastus Lateralis	1.36E-02	9.01E+00	1.49E+01	7.71E+00	6.13E-01	9.48E-03	5.13E-03	9.46E-03	9.17E-02
Vastus Medialis	9.74E-03	2.84E+00	4.82E+00	2.56E+00	3.23E-01	6.42E-03	3.18E-03	6.01E-03	6.89E-02

Table S7: Subject 2 - Anterior (+) Posterior (-) component of the muscle forces (Unit: N)

Load Instance	*1*	*2*	*3*	*4*	*5*	*6*	*7*	*8*	*9*
Timing (% gait cycle)	*0*	*9*	*17*	*24*	*31*	*40*	*50*	*58*	*66*
Gluteus Maximus Anterior	1.33E+00	4.44E+01	3.04E+01	3.02E+00	2.50E-01	-3.45E-01	-3.02E-02	-2.11E-02	5.17E-02
Gluteus Maximus Middle	2.02E-01	3.84E+00	-7.16E+00	-3.38E+00	-1.39E+00	-5.45E-02	-1.21E-02	-2.56E-02	-3.76E-02

Gluteus Maximus Posterior	-5.18E-01	-5.67E+00	-2.62E-02	-1.71E-02	-1.65E-02	-1.58E-02	-6.53E-03	-1.34E-02	-2.45E-01
Gluteus Medius Anterior	1.06E+01	4.19E+01	1.65E+02	1.17E+02	6.81E+01	4.30E+01	4.49E+01	1.68E+01	4.68E-02
Gluteus Medius Middle	8.51E+00	1.80E+01	3.96E+01	2.68E+01	1.26E+01	4.79E+00	1.05E+00	3.37E+00	5.64E-01
Gluteus Medius Posterior	8.30E+00	2.06E+01	1.57E+01	4.25E+00	-9.89E-01	-4.40E+00	-4.16E+00	-1.66E+00	1.70E+00
Gluteus Minimus Anterior	2.10E+00	2.97E+00	1.42E+01	1.29E+01	8.20E+00	5.17E+00	6.41E+00	3.94E+00	2.76E-01
Gluteus Minimus Middle	2.50E+00	2.88E+00	1.23E+01	9.32E+00	5.42E+00	3.03E+00	2.67E+00	2.25E+00	4.29E-01
Gluteus Minimus Posterior	3.36E+00	2.93E+00	1.01E+01	4.75E+00	2.09E+00	5.31E-01	-5.37E-01	3.64E-01	5.17E-01
Adductor Longus	-3.23E-02	-9.95E-03	4.37E-04	1.27E-03	2.54E-03	5.80E-03	4.76E-03	-4.68E-03	1.34E+00
Adductor Magnus Anterior	-2.86E-01	-4.01E-01	-7.68E-03	-4.02E-03	-2.98E-03	-2.40E-05	2.25E-03	-1.69E-03	-9.94E-01
Adductor Magnus Middle	-2.09E-01	-5.23E-01	-7.76E-03	-4.01E-03	-3.81E-03	-3.19E-03	-7.61E-04	-4.41E-03	-5.56E-01
Adductor Magnus Posterior	-1.67E-01	-1.17E+00	-1.13E-02	-5.12E-03	-4.12E-03	-2.21E-03	-1.79E-04	-1.69E-03	-1.50E-02
Adductor Brevis	-1.88E-01	-1.81E-01	-3.82E-03	-1.62E-03	-5.54E-04	2.36E-03	3.27E-03	2.40E-03	-2.93E-01
Iliacus	2.86E+00	1.05E-01	3.35E-01	1.76E+01	2.80E+01	6.48E+01	1.13E+02	8.49E+01	2.83E+01
Pectineus	2.22E-01	1.27E-01	1.34E-02	1.08E-02	1.73E-02	6.19E-02	3.20E-02	1.71E-01	7.47E-01
Piriformis	7.77E-03	-2.16E-01	-4.60E+00	-2.56E+00	-1.53E+00	-2.96E+00	-3.50E-01	-9.74E-01	-1.84E+00
Psoas	3.09E+00	1.00E-01	2.00E+00	2.30E+01	3.22E+01	7.67E+01	1.33E+02	9.63E+01	3.21E+01
Quadratus Femoris	-1.13E+00	-2.91E-01	-1.88E-02	-8.81E-03	-6.96E-03	-3.01E-03	2.29E-03	-5.79E-03	-4.38E+00
Gemelleus	-9.86E-01	-3.45E-01	-1.02E-01	-3.29E-02	-3.13E-02	-8.24E-02	-1.51E-02	-8.62E-02	-9.29E-01
Sartorius	-2.65E-01	-5.88E-02	-3.09E-01	-3.91E-01	-3.39E-01	-3.07E-01	-8.17E-01	-1.35E+00	-6.72E-01

Biceps Femoris Short Head	-1.86E+00	-7.02E-01	-2.35E+00	1.23E-01	-1.63E+00	-2.81E+00	-1.12E+01	-1.09E+01	-1.40E+01
Gastrocnemius Lateral	-1.21E+00	-1.44E-02	-1.99E-02	-1.47E-01	-1.44E+00	-5.70E+00	-1.23E+01	-9.43E+00	-6.49E-01
Gastrocnemius Medial	-4.71E+00	-2.51E-02	-2.52E-02	-7.64E-02	-3.47E+00	-2.36E+01	-5.51E+01	-4.21E+01	-1.02E+00
Tensor Fascia Latae	-5.33E-01	-5.23E-01	-8.64E+00	-6.68E+00	-3.16E+00	-1.73E+00	-4.23E+00	-4.99E+00	-1.15E+00
Vastus Intermedius	1.36E-02	6.08E+00	9.32E+00	4.61E+00	4.89E-01	8.92E-03	4.87E-03	8.00E-03	4.90E-02
Vastus Lateralis	2.85E-02	1.80E+01	3.02E+01	1.60E+01	1.28E+00	1.98E-02	1.07E-02	1.60E-02	9.62E-02
Vastus Medialis	2.05E-02	5.73E+00	9.87E+00	5.40E+00	6.78E-01	1.34E-02	6.70E-03	9.49E-03	4.40E-02

Table S8: Subject 2 - Superior (+) Inferior (-) component of the muscle forces (Unit: N)

Load Instance	*1*	*2*	*3*	*4*	*5*	*6*	*7*	*8*	*9*
percent	*0*	*9*	*17*	*24*	*31*	*40*	*50*	*58*	*66*
Gluteus Maximus Anterior	2.53E+00	9.93E+01	1.04E+02	2.03E+01	7.61E+00	3.87E+00	1.76E-01	3.58E-01	3.15E-01
Gluteus Maximus Middle	2.64E+00	1.20E+02	1.17E+02	2.21E+01	6.05E+00	1.72E-01	3.19E-02	8.48E-02	2.41E-01
Gluteus Maximus Posterior	1.00E+00	9.91E+00	3.75E-02	2.10E-02	1.86E-02	1.74E-02	8.02E-03	1.67E-02	3.60E-01
Gluteus Medius Anterior	5.84E+00	2.80E+01	1.56E+02	1.50E+02	1.15E+02	1.02E+02	1.48E+02	3.43E+01	5.46E-02
Gluteus Medius Middle	8.65E+00	2.11E+01	6.55E+01	6.70E+01	5.07E+01	4.49E+01	6.09E+01	2.39E+01	1.34E+00
Gluteus Medius Posterior	1.47E+01	4.32E+01	5.44E+01	3.95E+01	2.51E+01	2.43E+01	1.60E+01	1.18E+01	1.29E+01
Gluteus Minimus Anterior	1.11E+00	1.92E+00	1.32E+01	1.65E+01	1.38E+01	1.24E+01	2.14E+01	8.14E+00	3.20E-01
Gluteus Minimus Middle	1.95E+00	2.65E+00	1.58E+01	1.70E+01	1.38E+01	1.26E+01	1.94E+01	7.79E+00	7.23E-01

Gluteus Minimus Posterior	3.87E+00	3.93E+00	1.94E+01	1.45E+01	1.12E+01	1.13E+01	1.28E+01	4.47E+00	1.47E+00
Adductor Longus	6.70E-01	3.77E-01	2.13E-02	1.91E-02	2.54E-02	3.95E-02	2.51E-02	-3.72E-02	1.76E+01
Adductor Magnus Anterior	4.98E-01	6.94E-01	1.44E-02	1.02E-02	1.28E-02	1.80E-02	1.16E-02	3.19E-02	3.53E+00
Adductor Magnus Middle	6.57E-01	1.68E+00	2.74E-02	1.71E-02	2.08E-02	2.88E-02	1.68E-02	3.57E-02	2.68E+00
Adductor Magnus Posterior	1.02E+00	7.47E+00	8.43E-02	4.88E-02	5.32E-02	5.52E-02	2.56E-02	3.47E-02	1.57E-01
Adductor Brevis	6.37E-01	6.53E-01	1.78E-02	1.36E-02	1.78E-02	2.57E-02	1.63E-02	4.62E-02	4.30E+00
Iliacus	2.48E+00	9.73E-02	3.61E-01	2.23E+01	4.10E+01	1.10E+02	2.15E+02	1.39E+02	3.55E+01
Pectineus	7.89E-01	3.75E-01	3.08E-02	2.18E-02	3.30E-02	1.10E-01	5.37E-02	3.44E-01	1.68E+00
Piriformis	6.35E+00	2.53E+00	1.54E+01	4.71E+00	1.95E+00	2.74E+00	2.77E-01	1.06E+00	4.06E+00
Psoas	2.74E+00	9.21E-02	2.08E+00	2.81E+01	4.52E+01	1.24E+02	2.35E+02	1.54E+02	4.04E+01
Quadratus Femoris	5.36E-01	7.76E-02	-3.86E-03	-7.37E-03	-1.31E-02	-2.98E-02	-1.35E-02	-2.40E-02	-1.84E+00
Gemelleus	4.13E-01	9.08E-02	-4.30E-03	-1.06E-02	-1.76E-02	-7.13E-02	-1.72E-02	-6.08E-02	-1.92E-01
Sartorius	-1.76E+00	-2.32E-01	-1.29E+00	-2.09E+00	-2.35E+00	-2.45E+00	-4.34E+00	-3.68E+00	-1.30E+00
Biceps Femoris Short Head	-1.60E+01	-3.89E+00	-1.36E+01	8.78E-01	-1.45E+01	-2.79E+01	-7.96E+01	-4.44E+01	-4.36E+01
Gastrocnemius Lateral	-5.12E+00	-2.61E-02	-3.96E-02	-4.34E-01	-6.57E+00	-3.33E+01	-3.60E+01	-8.56E+00	-2.35E-01
Gastrocnemius Medial	-2.25E+01	-4.87E-02	-5.41E-02	-2.47E-01	-1.80E+01	-1.62E+02	-1.77E+02	-4.14E+01	-4.65E-01
Tensor Fascia Latae	-2.27E+00	-1.07E+00	-1.91E+01	-2.07E+01	-1.44E+01	-9.92E+00	-1.30E+01	-6.21E+00	-8.17E-01
Vastus Intermedius	-1.25E-01	-6.08E+01	-9.14E+01	-4.31E+01	-4.49E+00	-8.12E-02	-4.56E-02	-1.01E-01	-1.16E+00
Vastus Lateralis	-1.22E-01	-9.03E+01	-1.47E+02	-7.22E+01	-5.47E+00	-8.26E-02	-4.81E-02	-1.04E-01	-1.11E+00
Vastus Medialis	-1.26E-01	-4.15E+01	-6.93E+01	-3.47E+01	-4.12E+00	-7.99E-02	-4.30E-02	-9.71E-02	-1.24E+00

Table S9: Subject 2 - Medial (+) Lateral (-) component of the muscle forces (Unit: N)

Load Instance	*1*	*2*	*3*	*4*	*5*	*6*	*7*	*8*	*9*
percent	*0*	*9*	*17*	*24*	*31*	*40*	*50*	*58*	*66*
Gluteus Maximus Anterior	1.19E-01	9.76E+00	1.71E+01	3.62E+00	1.28E+00	7.04E-01	3.78E-02	1.27E-02	-7.99E-03
Gluteus Maximus Middle	-2.25E-01	-6.11E+00	-1.81E+00	-3.40E-01	-1.56E-01	1.69E-03	3.06E-03	-4.52E-03	-2.18E-02
Gluteus Maximus Posterior	2.36E-01	2.69E+00	1.23E-02	8.26E-03	8.91E-03	1.20E-02	7.56E-03	1.12E-02	1.65E-01
Gluteus Medius Anterior	5.10E-01	3.36E+00	2.62E+01	2.41E+01	1.48E+01	8.32E+00	4.05E+00	-2.44E+00	-6.40E-03
Gluteus Medius Middle	3.15E+00	8.50E+00	2.95E+01	2.96E+01	2.10E+01	1.75E+01	2.26E+01	5.25E+00	2.28E-01
Gluteus Medius Posterior	7.02E+00	2.32E+01	3.40E+01	2.57E+01	1.65E+01	1.68E+01	1.18E+01	5.64E+00	4.76E+00
Gluteus Minimus Anterior	6.66E-01	1.06E+00	6.70E+00	7.49E+00	5.55E+00	4.15E+00	5.81E+00	1.65E+00	6.64E-02
Gluteus Minimus Middle	1.05E+00	1.42E+00	8.45E+00	8.47E+00	6.31E+00	5.18E+00	7.22E+00	2.12E+00	1.85E-01
Gluteus Minimus Posterior	2.30E+00	2.44E+00	1.27E+01	9.26E+00	6.89E+00	6.76E+00	7.53E+00	1.93E+00	5.66E-01
Adductor Longus	3.28E-01	1.84E-01	1.02E-02	9.03E-03	1.18E-02	1.77E-02	1.06E-02	-1.68E-02	8.26E+00
Adductor Magnus Anterior	4.65E-01	6.93E-01	1.62E-02	1.25E-02	1.65E-02	2.40E-02	1.52E-02	4.08E-02	4.16E+00
Adductor Magnus Middle	2.12E-01	5.47E-01	8.95E-03	5.75E-03	7.30E-03	1.07E-02	6.49E-03	1.46E-02	1.06E+00
Adductor Magnus Posterior	2.33E-01	1.69E+00	1.87E-02	1.10E-02	1.23E-02	1.31E-02	6.27E-03	9.04E-03	4.09E-02

Adductor Brevis	5.49E-01	5.88E-01	1.70E-02	1.33E-02	1.73E-02	2.46E-02	1.49E-02	4.23E-02	3.88E+00
Iliacus	-4.21E-01	-1.94E-02	-6.55E-02	-3.28E+00	-5.36E+00	-1.76E+01	-4.68E+01	-2.53E+01	-6.91E+00
Pectineus	7.13E-01	3.41E-01	2.75E-02	1.85E-02	2.66E-02	8.13E-02	3.56E-02	2.46E-01	1.31E+00
Piriformis	4.47E+00	2.03E+00	1.52E+01	5.36E+00	2.51E+00	4.37E+00	5.36E-01	1.28E+00	3.30E+00
Psoas	3.13E-01	4.80E-03	3.00E-02	4.22E-01	5.66E-01	-4.07E+00	-2.38E+01	-7.15E+00	-9.08E-01
Quadratus Femoris	1.31E+00	3.51E-01	2.53E-02	1.59E-02	2.04E-02	4.11E-02	1.97E-02	5.11E-02	1.13E+01
Gemelleus	1.27E+00	4.45E-01	1.29E-01	4.21E-02	4.26E-02	1.35E-01	3.15E-02	1.44E-01	1.33E+00
Sartorius	-5.15E-02	-7.04E-03	-3.89E-02	-6.17E-02	-6.87E-02	-7.12E-02	-1.28E-01	-1.19E-01	-4.80E-02
Biceps Femoris Short Head	-1.75E+00	-4.38E-01	-1.53E+00	9.72E-02	-1.59E+00	-3.06E+00	-8.81E+00	-5.22E+00	-5.57E+00
Gastrocnemius Lateral	4.39E-01	2.85E-03	4.16E-03	4.01E-02	5.57E-01	2.73E+00	3.33E+00	1.40E+00	5.90E-02
Gastrocnemius Medial	2.40E+00	6.47E-03	6.93E-03	2.82E-02	1.90E+00	1.66E+01	2.02E+01	7.93E+00	1.81E-01
Tensor Fascia Latae	2.93E-01	1.40E-01	2.48E+00	2.66E+00	1.86E+00	1.29E+00	1.68E+00	8.81E-01	1.47E-01
Vastus Intermedius	1.15E-02	5.14E+00	7.81E+00	3.84E+00	4.13E-01	7.59E-03	4.05E-03	7.99E-03	8.54E-02
Vastus Lateralis	1.57E-02	1.04E+01	1.71E+01	8.87E+00	7.05E-01	1.09E-02	5.90E-03	1.09E-02	1.05E-01
Vastus Medialis	1.12E-02	3.27E+00	5.54E+00	2.95E+00	3.71E-01	7.38E-03	3.66E-03	6.91E-03	7.92E-02

Table S10: Subject 3 - Anterior (+) Posterior (-) component of the muscle forces (Unit: N)

Load Instance	1	2	3	4	5	6	7	8	9
percent	0	9	19	27	35	41	48	57	66
Gluteus Maximus Anterior	2.77E+00	9.24E+01	6.32E+01	6.28E+00	5.20E-01	-7.18E-01	-6.28E-02	-4.39E-02	1.08E-01
Gluteus Maximus Middle	4.19E-01	7.99E+00	-1.49E+01	-7.03E+00	-2.89E+00	-1.13E-01	-2.52E-02	-5.33E-02	-7.83E-02
Gluteus Maximus Posterior	-1.08E+00	-1.18E+01	-5.45E-02	-3.55E-02	-3.43E-02	-3.28E-02	-1.36E-02	-2.78E-02	-5.10E-01
Gluteus Medius Anterior	2.20E+01	8.72E+01	3.44E+02	2.43E+02	1.42E+02	8.93E+01	9.35E+01	3.49E+01	9.74E-02
Gluteus Medius Middle	1.77E+01	3.73E+01	8.24E+01	5.57E+01	2.63E+01	9.96E+00	2.17E+00	7.02E+00	1.17E+00
Gluteus Medius Posterior	1.73E+01	4.28E+01	3.27E+01	8.83E+00	-2.06E+00	-9.16E+00	-8.65E+00	-3.45E+00	3.53E+00
Gluteus Minimus Anterior	4.36E+00	6.17E+00	2.95E+01	2.68E+01	1.70E+01	1.08E+01	1.33E+01	8.19E+00	5.74E-01
Gluteus Minimus Middle	5.21E+00	5.99E+00	2.56E+01	1.94E+01	1.13E+01	6.30E+00	5.56E+00	4.67E+00	8.93E-01
Gluteus Minimus Posterior	6.99E+00	6.10E+00	2.11E+01	9.89E+00	4.34E+00	1.11E+00	-1.12E+00	7.58E-01	1.07E+00
Adductor Longus	-6.72E-02	-2.07E-02	9.10E-04	2.65E-03	5.28E-03	1.21E-02	9.89E-03	-9.74E-03	2.78E+00
Adductor Magnus Anterior	-5.95E-01	-8.34E-01	-1.60E-02	-8.36E-03	-6.20E-03	-4.99E-05	4.68E-03	-3.52E-03	-2.07E+00
Adductor Magnus Middle	-4.34E-01	-1.09E+00	-1.61E-02	-8.34E-03	-7.92E-03	-6.64E-03	-1.58E-03	-9.16E-03	-1.16E+00
Adductor Magnus Posterior	-3.47E-01	-2.42E+00	-2.35E-02	-1.06E-02	-8.57E-03	-4.60E-03	-3.73E-04	-3.51E-03	-3.11E-02
Adductor Brevis	-3.90E-01	-3.76E-01	-7.95E-03	-3.36E-03	-1.15E-03	4.92E-03	6.81E-03	4.99E-03	-6.09E-01
Iliacus	5.95E+00	2.19E-01	6.97E-01	3.66E+01	5.83E+01	1.35E+02	2.36E+02	1.77E+02	5.88E+01
Pectineus	4.62E-01	2.63E-01	2.79E-02	2.25E-02	3.59E-02	1.29E-01	6.67E-02	3.55E-01	1.55E+00

Piriformis	1.62E-02	-4.48E-01	-9.56E+00	-5.33E+00	-3.19E+00	-6.15E+00	-7.27E-01	-2.02E+00	-3.83E+00
Psoas	6.43E+00	2.09E-01	4.15E+00	4.79E+01	6.69E+01	1.60E+02	2.77E+02	2.00E+02	6.68E+01
Quadratus Femoris	-2.35E+00	-6.06E-01	-3.91E-02	-1.83E-02	-1.45E-02	-6.26E-03	4.77E-03	-1.20E-02	-9.11E+00
Gemelleus	-2.05E+00	-7.19E-01	-2.13E-01	-6.85E-02	-6.51E-02	-1.71E-01	-3.14E-02	-1.79E-01	-1.93E+00
Sartorius	-5.52E-01	-1.22E-01	-6.43E-01	-8.14E-01	-7.05E-01	-6.39E-01	-1.70E+00	-2.80E+00	-1.40E+00
Biceps Femoris Short Head	-3.87E+00	-1.46E+00	-4.88E+00	2.55E-01	-3.39E+00	-5.84E+00	-2.32E+01	-2.26E+01	-2.92E+01
Gastrocnemius Lateral	-2.52E+00	-3.00E-02	-4.14E-02	-3.06E-01	-3.00E+00	-1.19E+01	-2.55E+01	-1.96E+01	-1.35E+00
Gastrocnemius Medial	-9.80E+00	-5.21E-02	-5.25E-02	-1.59E-01	-7.22E+00	-4.91E+01	-1.15E+02	-8.75E+01	-2.13E+00
Tensor Fascia Latae	-1.11E+00	-1.09E+00	-1.80E+01	-1.39E+01	-6.57E+00	-3.61E+00	-8.80E+00	-1.04E+01	-2.39E+00
Vastus Intermedius	2.83E-02	1.26E+01	1.94E+01	9.59E+00	1.02E+00	1.85E-02	1.01E-02	1.66E-02	1.02E-01
Vastus Lateralis	5.93E-02	3.75E+01	6.28E+01	3.34E+01	2.67E+00	4.13E-02	2.22E-02	3.33E-02	2.00E-01
Vastus Medialis	4.26E-02	1.19E+01	2.05E+01	1.12E+01	1.41E+00	2.79E-02	1.39E-02	1.97E-02	9.15E-02

Table S11: Subject 3 - Superior (+) Inferior (-) component of the muscle forces (Unit: N)

Load Instance	1	2	3	4	5	6	7	8	9
percent	0	9	19	27	35	41	48	57	66
Gluteus Maximus Anterior	5.27E+00	2.07E+02	2.16E+02	4.22E+01	1.58E+01	8.04E+00	3.66E-01	7.44E-01	6.56E-01
Gluteus Maximus Middle	5.50E+00	2.50E+02	2.44E+02	4.60E+01	1.26E+01	3.57E-01	6.63E-02	1.76E-01	5.02E-01
Gluteus Maximus Posterior	2.08E+00	2.06E+01	7.80E-02	4.37E-02	3.86E-02	3.62E-02	1.67E-02	3.47E-02	7.48E-01

Gluteus Medius Anterior	1.21E+01	5.82E+01	3.24E+02	3.13E+02	2.39E+02	2.12E+02	3.07E+02	7.14E+01	1.14E-01
Gluteus Medius Middle	1.80E+01	4.39E+01	1.36E+02	1.39E+02	1.05E+02	9.34E+01	1.27E+02	4.96E+01	2.78E+00
Gluteus Medius Posterior	3.06E+01	8.98E+01	1.13E+02	8.21E+01	5.23E+01	5.06E+01	3.33E+01	2.45E+01	2.68E+01
Gluteus Minimus Anterior	2.31E+00	3.98E+00	2.74E+01	3.43E+01	2.88E+01	2.58E+01	4.45E+01	1.69E+01	6.65E-01
Gluteus Minimus Middle	4.06E+00	5.51E+00	3.29E+01	3.53E+01	2.87E+01	2.61E+01	4.04E+01	1.62E+01	1.50E+00
Gluteus Minimus Posterior	8.05E+00	8.17E+00	4.05E+01	3.01E+01	2.34E+01	2.35E+01	2.67E+01	9.29E+00	3.07E+00
Adductor Longus	1.39E+00	7.84E-01	4.43E-02	3.97E-02	5.28E-02	8.22E-02	5.23E-02	-7.74E-02	3.65E+01
Adductor Magnus Anterior	1.04E+00	1.44E+00	3.00E-02	2.12E-02	2.66E-02	3.75E-02	2.41E-02	6.64E-02	7.35E+00
Adductor Magnus Middle	1.37E+00	3.49E+00	5.70E-02	3.56E-02	4.33E-02	5.99E-02	3.49E-02	7.42E-02	5.57E+00
Adductor Magnus Posterior	2.12E+00	1.55E+01	1.75E-01	1.02E-01	1.11E-01	1.15E-01	5.33E-02	7.21E-02	3.26E-01
Adductor Brevis	1.32E+00	1.36E+00	3.71E-02	2.84E-02	3.69E-02	5.35E-02	3.40E-02	9.62E-02	8.95E+00
Iliacus	5.16E+00	2.02E-01	7.51E-01	4.65E+01	8.53E+01	2.29E+02	4.47E+02	2.89E+02	7.37E+01
Pectineus	1.64E+00	7.80E-01	6.41E-02	4.53E-02	6.87E-02	2.29E-01	1.12E-01	7.16E-01	3.49E+00
Piriformis	1.32E+01	5.25E+00	3.20E+01	9.79E+00	4.06E+00	5.70E+00	5.77E-01	2.21E+00	8.45E+00
Psoas	5.71E+00	1.91E-01	4.33E+00	5.85E+01	9.40E+01	2.57E+02	4.88E+02	3.21E+02	8.40E+01
Quadratus Femoris	1.11E+00	1.61E-01	-8.03E-03	-1.53E-02	-2.73E-02	-6.20E-02	-2.81E-02	-4.99E-02	-3.84E+00
Gemelleus	8.59E-01	1.89E-01	-8.95E-03	-2.20E-02	-3.65E-02	-1.48E-01	-3.59E-02	-1.26E-01	-4.00E-01
Sartorius	-3.67E+00	-4.82E-01	-2.69E+00	-4.34E+00	-4.89E+00	-5.10E+00	-9.03E+00	-7.66E+00	-2.71E+00
Biceps Femoris Short Head	-3.32E+01	-8.09E+00	-2.84E+01	1.83E+00	-3.01E+01	-5.81E+01	-1.66E+02	-9.23E+01	-9.06E+01
Gastrocnemius	-1.06E+01	-5.42E-02	-8.24E-02	-9.02E-01	-1.37E+01	-6.92E+01	-7.49E+01	-1.78E+01	-4.89E-01

Lateral									
Gastrocnemius Medial	-4.67E+01	-1.01E-01	-1.12E-01	-5.14E-01	-3.74E+01	-3.37E+02	-3.69E+02	-8.62E+01	-9.68E-01
Tensor Fascia Latae	-4.72E+00	-2.22E+00	-3.97E+01	-4.30E+01	-2.99E+01	-2.06E+01	-2.71E+01	-1.29E+01	-1.70E+00
Vastus Intermedius	-2.60E-01	-1.26E+02	-1.90E+02	-8.97E+01	-9.33E+00	-1.69E-01	-9.47E-02	-2.10E-01	-2.42E+00
Vastus Lateralis	-2.55E-01	-1.88E+02	-3.06E+02	-1.50E+02	-1.14E+01	-1.72E-01	-1.00E-01	-2.16E-01	-2.30E+00
Vastus Medialis	-2.61E-01	-8.63E+01	-1.44E+02	-7.21E+01	-8.57E+00	-1.66E-01	-8.95E-02	-2.02E-01	-2.57E+00

Table S12: Subject 3 - Medial (+) Lateral (-) component of the muscle forces (Unit: N)

Load Instance	1	2	3	4	5	6	7	8	9
percent	0	9	19	27	35	41	48	57	66
Gluteus Maximus Anterior	2.47E-01	2.03E+01	3.57E+01	7.52E+00	2.66E+00	1.46E+00	7.86E-02	2.64E-02	-1.66E-02
Gluteus Maximus Middle	-4.69E-01	-1.27E+01	-3.76E+00	-7.07E-01	-3.24E-01	3.52E-03	6.36E-03	-9.41E-03	-4.54E-02
Gluteus Maximus Posterior	4.90E-01	5.60E+00	2.56E-02	1.72E-02	1.85E-02	2.49E-02	1.57E-02	2.33E-02	3.42E-01
Gluteus Medius Anterior	1.06E+00	7.00E+00	5.44E+01	5.00E+01	3.08E+01	1.73E+01	8.42E+00	-5.07E+00	-1.33E-02
Gluteus Medius Middle	6.54E+00	1.77E+01	6.13E+01	6.15E+01	4.36E+01	3.65E+01	4.70E+01	1.09E+01	4.74E-01
Gluteus Medius Posterior	1.46E+01	4.83E+01	7.07E+01	5.35E+01	3.42E+01	3.50E+01	2.45E+01	1.17E+01	9.91E+00
Gluteus Minimus Anterior	1.39E+00	2.21E+00	1.39E+01	1.56E+01	1.15E+01	8.64E+00	1.21E+01	3.43E+00	1.38E-01
Gluteus Minimus Middle	2.19E+00	2.96E+00	1.76E+01	1.76E+01	1.31E+01	1.08E+01	1.50E+01	4.41E+00	3.86E-01
Gluteus Minimus Posterior	4.78E+00	5.07E+00	2.64E+01	1.92E+01	1.43E+01	1.41E+01	1.57E+01	4.02E+00	1.18E+00

Adductor Longus	6.82E-01	3.83E-01	2.13E-02	1.88E-02	2.46E-02	3.68E-02	2.20E-02	-3.49E-02	1.72E+01
Adductor Magnus Anterior	9.66E-01	1.44E+00	3.36E-02	2.59E-02	3.44E-02	4.98E-02	3.16E-02	8.48E-02	8.65E+00
Adductor Magnus Middle	4.41E-01	1.14E+00	1.86E-02	1.20E-02	1.52E-02	2.22E-02	1.35E-02	3.03E-02	2.21E+00
Adductor Magnus Posterior	4.84E-01	3.52E+00	3.90E-02	2.28E-02	2.55E-02	2.73E-02	1.30E-02	1.88E-02	8.50E-02
Adductor Brevis	1.14E+00	1.22E+00	3.54E-02	2.77E-02	3.61E-02	5.11E-02	3.10E-02	8.79E-02	8.08E+00
Iliacus	-8.76E-01	-4.03E-02	-1.36E-01	-6.83E+00	-1.12E+01	-3.66E+01	-9.74E+01	-5.27E+01	-1.44E+01
Pectineus	1.48E+00	7.09E-01	5.71E-02	3.85E-02	5.54E-02	1.69E-01	7.41E-02	5.12E-01	2.72E+00
Piriformis	9.30E+00	4.22E+00	3.15E+01	1.12E+01	5.21E+00	9.09E+00	1.11E+00	2.65E+00	6.85E+00
Psoas	6.52E-01	9.97E-03	6.24E-02	8.77E-01	1.18E+00	-8.45E+00	-4.94E+01	-1.49E+01	-1.89E+00
Quadratus Femoris	2.73E+00	7.30E-01	5.26E-02	3.30E-02	4.24E-02	8.56E-02	4.10E-02	1.06E-01	2.35E+01
Gemelleus	2.64E+00	9.25E-01	2.67E-01	8.75E-02	8.86E-02	2.81E-01	6.54E-02	2.99E-01	2.76E+00
Sartorius	-1.07E-01	-1.46E-02	-8.09E-02	-1.28E-01	-1.43E-01	-1.48E-01	-2.67E-01	-2.48E-01	-9.98E-02
Biceps Femoris Short Head	-3.65E+00	-9.12E-01	-3.19E+00	2.02E-01	-3.31E+00	-6.36E+00	-1.83E+01	-1.09E+01	-1.16E+01
Gastrocnemius Lateral	9.13E-01	5.93E-03	8.66E-03	8.34E-02	1.16E+00	5.68E+00	6.92E+00	2.92E+00	1.23E-01
Gastrocnemius Medial	4.98E+00	1.34E-02	1.44E-02	5.86E-02	3.95E+00	3.46E+01	4.21E+01	1.65E+01	3.76E-01
Tensor Fascia Latae	6.10E-01	2.91E-01	5.16E+00	5.54E+00	3.87E+00	2.69E+00	3.49E+00	1.83E+00	3.06E-01
Vastus Intermedius	2.38E-02	1.07E+01	1.63E+01	7.98E+00	8.59E-01	1.58E-02	8.42E-03	1.66E-02	1.78E-01
Vastus Lateralis	3.26E-02	2.15E+01	3.56E+01	1.84E+01	1.47E+00	2.27E-02	1.23E-02	2.26E-02	2.19E-01
Vastus Medialis	2.33E-02	6.80E+00	1.15E+01	6.13E+00	7.72E-01	1.53E-02	7.61E-03	1.44E-02	1.65E-01
